# COVID-19 Transmission Potential and Non-Pharmaceutical Interventions in Maine During the COVID-19 Pandemic

**DOI:** 10.3390/pathogens14090893

**Published:** 2025-09-05

**Authors:** Ina Sze-Ting Lee, Sylvia K. Ofori, Doyinsola A. Babatunde, Emmanuel A. Akowuah, Kin On Kwok, Gerardo Chowell, Isaac Chun-Hai Fung

**Affiliations:** 1 School of Public Health, Boston University, Boston, MA 02118, USA; inaleect@bu.edu; 2 Department of Biostatistics, Epidemiology and Environmental Health Sciences, Jiann-Ping Hsu College of Public Health, Georgia Southern University, Statesboro, GA 30460, USA; kesewa15@gmail.com (S.K.O.); doyinsolababatunde@gmail.com (D.A.B.); 3 Independent Researcher, Nashville, TN 37122, USA; 4 Independent Researcher, Houston, TX 75201, USA; 5 Department of Public Health, College of Pharmacy and Health Sciences, Belmont University, Nashville, TN 37212, USA; emmanuel.akowuah@belmont.edu; 6 J.C. School of Public Health and Primary Care, The Chinese University of Hong Kong, Hong Kong SAR, China; k.kwok@imperial.ac.uk; 7 Hong Kong Institute of Asia-Pacific Studies, The Chinese University of Hong Kong, Hong Kong SAR, China; 8 Department of Infectious Disease Epidemiology, School of Public Health, Imperial College London, Wood Lane, White City, London W12 0BZ, UK; 9 Department of Population Health Sciences, Georgia State University, Atlanta, GA 30303, USA; gchowell@gsu.edu

**Keywords:** COVID-19, epidemiology, non-pharmaceutical interventions, reproduction number, time series analysis, United States

## Abstract

The study aimed to evaluate regional variation in SARS-CoV-2 transmission and assess associations between public health interventions and the time-varying reproduction number (Rt) across Maine from January 2020 to February 2023. Daily confirmed COVID-19 case counts were adjusted for reporting anomalies and delays using deconvolution. Infection counts were estimated by applying a Poisson-distributed multiplier of 4 to account for underreporting. Rt was estimated using *EpiEstim* with a 7-day sliding window from January 2020 through February 2023. The analysis of associations between Rt and public health interventions was limited to 2020, concluding just before COVID-19 vaccines became available in Maine in December 2020. *EpiEstim* was parameterized with an Omicron-specific serial interval distribution (main analysis) and an early-pandemic serial interval distribution (sensitivity analysis). Maine experienced four major COVID-19 waves. Rt values fluctuated but remained close to 1 at both the statewide and district levels. No statistically significant changes in Rt were observed in association with any interventions implemented in 2020. Our findings underscore the challenges of quantifying intervention impacts in rural settings, where low incidence and sparse data can obscure the effects of interventions. This highlights the need for enhanced surveillance tools tailored to the unique constraints of rural public health contexts.

## 1. Introduction

The coronavirus disease 2019 (COVID-19) pandemic posed a significant threat to global health [1]. In the United States, early transmission was concentrated in large metropolitan areas, including major cities in the Northeast [2]. Among the six New England states, Maine reported some of the lowest confirmed COVID-19 case rates in the United States during 2020 [3], a trend attributed to its early implementation of non-pharmaceutical interventions (NPIs) and strong public health coordination.

Maine provides a particularly relevant case study for understanding COVID-19 dynamics in rural settings. It is among the most rural states in the United States [4], with a population of approximately 1.4 million and nearly half of the land area largely uninhabited [4,5]. Approximately 40% of the population resides in the state’s 11 rural counties [4], which face unique challenges in accessing healthcare [6]. Although its low population density initially contributed to slower transmission [7], rural areas were disproportionately affected by the erosion of essential health services, financial pressures on hospitals, and shortages in the healthcare workforce [8]. Maine also has the oldest population in the United States, with a median age of 44.8 [9], further elevating the risk of severe COVID-19 outcomes, especially among older adults who are more vulnerable to serious complications from the disease [10].

Since 2008, the Maine Center for Disease Control and Prevention has organized the state into eight public health districts to improve the coordination and delivery of public health services. These districts divide the state’s 16 counties based on population size, geography, and hospital service areas [11]. Despite these structural efforts, rural and sparsely populated areas have continued to face persistent disparities in healthcare access, complicating COVID-19 testing, treatment, and vaccine distribution [12,13]. While multiple studies have assessed the effectiveness of NPIs in urban centers and high-incidence areas [14,15,16,17], fewer have examined their impact in rural regions like Maine, where data sparsity and low case counts complicate real-time assessment.

To characterize SARS-CoV-2 transmission dynamics in a predominantly rural setting, we estimated the time-varying reproduction number (Rt) for Maine, where Rt represents the expected number of secondary cases caused by an infected individual at a given time. The analysis had two primary objectives: (1) to assess regional variation in the transmission potential of SARS-CoV-2 across Maine’s public health districts and counties from January 2020 to February 2023; and (2) to examine the association between NPIs and Rt within these districts and counties during 2020, prior to the availability of COVID-19 vaccines in Maine.

## 2. Materials and Methods

### 2.1. Data Sources

Data were obtained from the COVID-19 Unified Dataset, compiled by the Johns Hopkins University Center for Systems Science and Engineering (CSSE) and made available through their GitHub repository, CSSEGISandData [18,19]. This dataset covers the period from 22 January 2020 to 9 March 2023, and includes daily confirmed incident case counts at both the state and county levels. County population estimates were obtained from the United States Census Bureau [20,21]. Information on policy measures implemented in Maine in response to the COVID-19 pandemic, spanning March through December 2020, was obtained from the Office of the Governor of the State of Maine (Table S1).

### 2.2. Unit of Analysis

**Figure 1:**
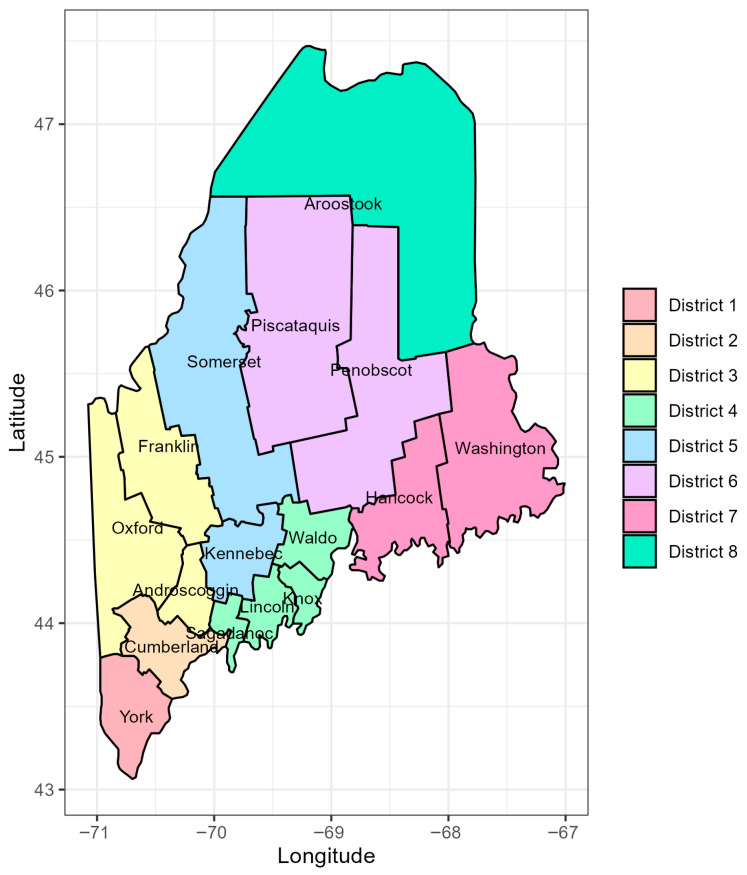
Grouping of Maine’s 16 counties into public health districts: District 1 (York); District 2 (Cumberland); District 3 (Androscoggin, Franklin, and Oxford); District 4 (Knox, Lincoln, Sagadahoc, and Waldo); District 5 (Kennebec and Somerset); District 6 (Penobscot and Piscataquis); District 7 (Hancock and Washington); and District 8 (Aroostook).

In this study, Maine’s 16 counties were grouped into eight public health districts, as defined by the Maine Legislature in collaboration with the Maine Department of Health and Human Services [11]. District 1 includes York County; District 2 includes Cumberland County; District 3 includes Androscoggin, Franklin, and Oxford counties; District 4 includes Knox, Lincoln, Sagadahoc, and Waldo counties; District 5 includes Kennebec and Somerset counties; District 6 includes Penobscot and Piscataquis counties; District 7 includes Hancock and Washington counties; and District 8 includes Aroostook County (Figure 1).

To evaluate whether aggregating counties into public health districts obscured intra-district heterogeneity in transmission dynamics or policy impacts, we conducted a sensitivity analysis at the county level. All steps described in Section 2.3 and Section 2.4 were repeated using each of Maine’s 16 counties as the unit of analysis, rather than the eight public health districts. County-level Rt estimates and percentage changes during policy periods were compared with district-level results to assess the robustness of spatial inferences.

### 2.3. Data Cleaning and Deconvolution

To estimate Rt, negative incident case counts were first corrected. Additionally, adjustments were made for the weekend effect, during which no cases were reported, to reduce artificial fluctuations in Rt. Negative values were identified and imputed using a local average of data points from three days before to three days after the corresponding date, resulting in a seven-day imputation window. Zero values reported during weekends and holidays were imputed using the same method. For data recorded before 16 March 2020 (the date marking the beginning of significant COVID-19 case reporting in Maine), zero case counts were not imputed, as they were assumed to reflect true zeros. Imputation near the end of the dataset was performed by averaging available surrounding values while accounting for boundary conditions. These methods were applied consistently across county-level data to ensure uniform corrections and enable the reliable estimation of infection trends and Rt.

Using the R package *incidental* (version 0.1), deconvolution was performed on the corrected incident case data to estimate infection dates by accounting for the delay between infection and case reporting. To make this adjustment, we applied the package’s default COVID-19 infection-to-case-report time-lag distribution (mean = 12.75 days, SD = 6.07 days), which is based on a 2020 dataset from Florida [22]. The package implements an empirical Bayes method to fit a regularized likelihood model on a spline basis, which reduces noise and smooths the deconvoluted case count curve. We applied a sequence from 40 to 60, in increments of 2, as the degrees of freedom input in the *dof_grid* parameter, instead of the default range of 6 to 20, allowing for greater flexibility in spline fitting and supporting a sensitivity analysis across spline configurations. To estimate daily infection counts, 1000 deconvoluted time series were generated. A Poisson-distributed multiplier of 4 was applied to each to account for the underreporting of asymptomatic and mildly symptomatic infections [23,24], resulting in 10,000 time series of estimated daily infections. The median and 95% credible interval (CrI) were then calculated from these time series. Additional sensitivity analyses were performed using multipliers of 3.4 and 4.7, corresponding to the United States CDC’s estimated lower and upper bounds for the number of infections per reported case [25].

### 2.4. Rt Estimation

Rt estimates were derived using the instantaneous reproduction number method, introduced by Cori et al. [26] and implemented in the R package *EpiEstim*. Rt is defined as the ratio of new cases at a given time to the total infectiousness of all infected individuals at that time. An Rt value greater than 1 indicates that transmission is increasing, while a value below 1 indicates that it is declining. For this analysis, we specified a prior distribution with a mean of 2 and a standard deviation of 2.

Temporal trends in Rt were analyzed using a 7-day sliding window from 22 January 2020, the start of the COVID-19 Unified Dataset, to 14 February 2023, the day before Maine transitioned from daily to weekly reporting. The main analysis used Omicron-specific serial interval distribution parameters (mean = 2.9 days, SD = 1.64 days) [27]. A sensitivity analysis was also conducted using an early-pandemic serial interval distribution (mean = 4.6 days, SD = 5.55 days) [28,29], along with an alternative prior distribution for Rt (mean = 5, SD = 5). For each Rt estimate within each sliding time window, 10 values were sampled from the posterior distribution of Rt for each of the 10,000 time series of estimated daily infections. From these, the median Rt and its 95% CrI were calculated.

We further evaluated changes in transmission across time periods defined by major COVID-19 policy measures introduced in 2020. This analysis was restricted to 22 January through 15 December 2020, ending just before COVID-19 vaccines became available in Maine to avoid confounding from gradual vaccination rollout to the public that could influence Rt independently of NPIs. Seven non-overlapping time windows were defined based on the timing of major COVID-19 policy measures (Table 1). For each period, the median Rt and its 95% CrI were calculated. To estimate percentage changes in Rt between successive time windows, we drew 10 Rt samples from the posterior distribution of each of the 10,000 infection time series, resulting in 100,000 Rt samples per time window. These samples were paired across successive time windows to compute 100,000 percentage changes. The median and 95% CrI of the resulting percentage change distribution were then derived empirically.

### 2.5. Statistical Software

All statistical analyses were performed using R version 4.4.1 (R Core Team, R Foundation for Statistical Computing, Vienna, Austria, 2024). All Rt estimates were obtained using the *EpiEstim* package (version 2.2.4).

## 3. Results

**Figure 2:**
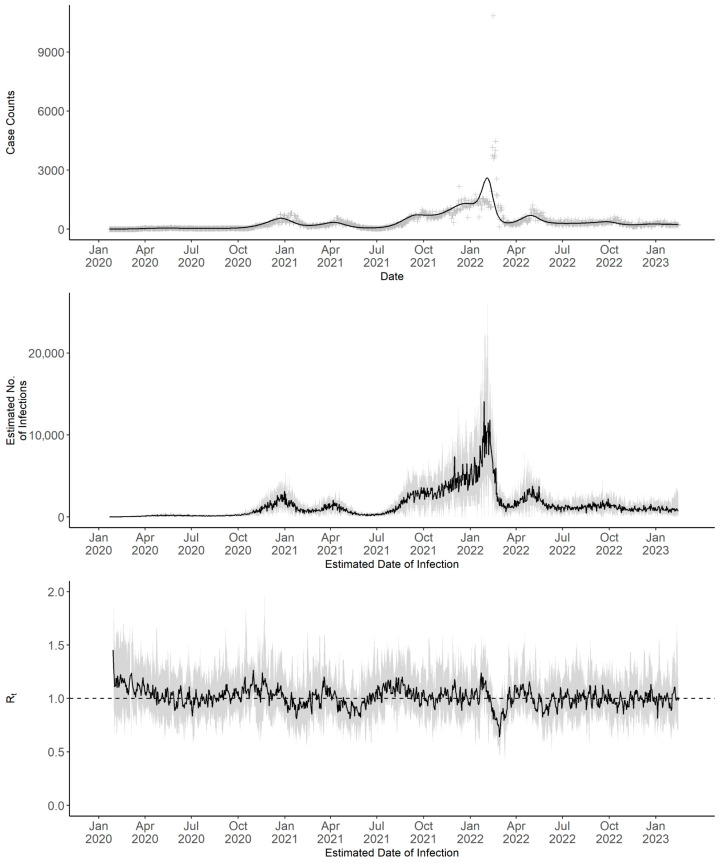
Daily observed incident case counts, deconvoluted incident case counts, estimated infection counts, and 7-day sliding window Rt with corresponding 95% CrI for Maine statewide from 22 January 2020 to 14 February 2023. In the upper panel, observed case counts are shown using grey crosses, and deconvoluted case counts are shown as a black line in the upper panel. In the middle panel, the median estimated number of new infections (black line), with corresponding 95% CrI (shaded area), is shown in the middle panel. In the lower panel, the median 7-day sliding window Rt (black line), with corresponding 95% CrI (shaded area), is shown in the lower panel.

The deconvoluted daily incident case counts, daily estimated number of infections, and 7-day sliding window Rt for Maine from 22 January 2020 to 14 February 2023 are shown in Figure 2. During this study period, Maine experienced four distinct waves of COVID-19 case surges. The first wave occurred between November 2020 and February 2021. This was followed by a second, smaller wave that peaked in April. The third and most substantial wave occurred between October 2021 and March 2022. Finally, a fourth, modest and transient wave was observed in April and May 2022. The deconvolution algorithm identified the best-fit deconvoluted incident case count curve with a degree of freedom of 56 in all time series, except in District 8, which had a degree of freedom of 54 (Table S2).

The 7-day sliding window Rt showed an initial spike in early 2020, followed by a relatively stable period with fluctuations around 1, despite a notable dip below 1 between January and April 2022. To test the sensitivity of our methodology, we repeated the 7-day sliding window analysis under alternative model specifications (Figures S1–S4). These included specifying the default range of degrees of freedom for smoothing (degrees of freedom from 6 to 20, in increments of 2; Figure S1), applying the default prior Rt distribution with a mean of 5 and a standard deviation of 5 (Figure S2), and applying an early-pandemic serial interval distribution (mean 4.6 days, SD 5.55 days) along with prior Rt distributions with means of 2 or 5 (SD 2 or 5, respectively) (Figures S3 and S4). Although models using the early-pandemic serial interval produced a higher initial Rt and a more rapid decline in early 2020, the estimated Rt over time was similar across all scenarios. This demonstrates that our Rt results are robust and not sensitive to changes in parametric specifications.

Infection counts estimated with multipliers of 3.4 and 4.7 differed in scale from those based on the multiplier of 4 used in the primary analysis but exhibited identical temporal patterns (Figure S5). The timing and shape of COVID-19 case surges in Maine were consistent across the three multiplier values. Corresponding Rt estimates remained aligned across these values, with 95% CrIs overlapping for nearly the entire study period.

**Figure 3:**
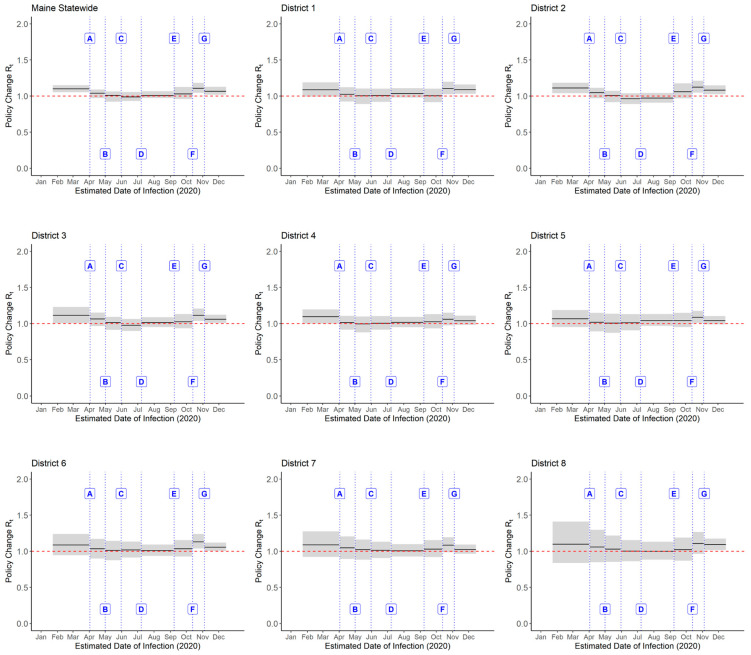
Changes in Rt following major policy interventions for Maine statewide and its eight public health districts in 2020. Labels: A—Stay Healthy at Home Directive (2 April 2020); B—Re-opening (Stage 1) (1 May 2020); C—Gradual Easing of Restrictions (31 May 2020); D—Face Covering Mandate (Certain Businesses) (8 July 2020); E—School Re-opening (8 September 2020); F—Re-opening (Stage 4) (13 October 2020); G—Face Covering Mandate (All Public Settings) (4 November 2020). Some periods, such as the initial lockdown (A) and school reopening (E), showed more pronounced directional changes in certain districts, as reflected in the median Rt estimates.

**Figure 4:**
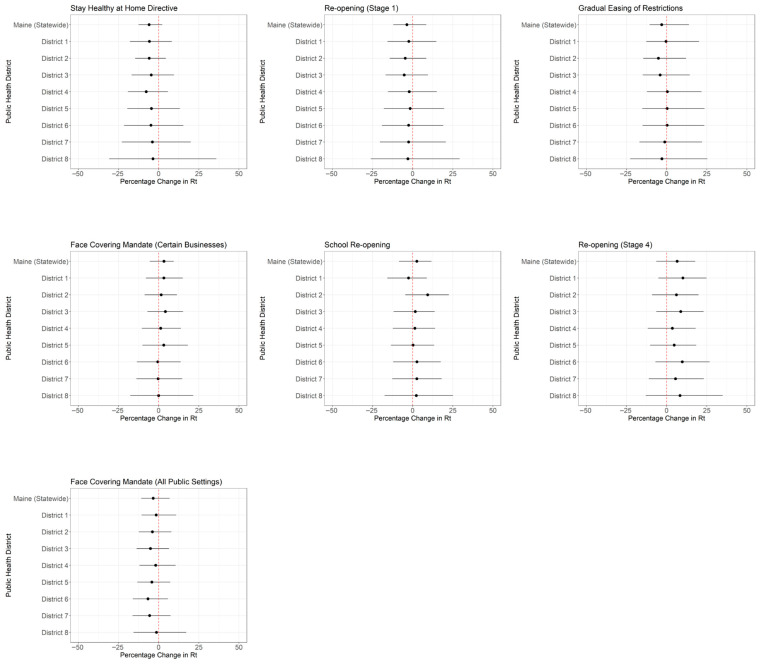
Median percentage change and 95% CrI of Rt estimates associated with policy changes, for Maine statewide and its eight public health districts. The *x*-axis represents the percentage change, with increases in Rt (positive values) shown to the right of the vertical dotted line at zero and decreases in Rt (negative values) shown to the left. While the 95% CrIs crossed zero for all comparisons, the initial Stay-at-Home directive and school reopening exhibited larger shifts, suggesting potential directional impacts.

The Maine government implemented a series of NPIs in 2020, spanning seven major policy periods. Following these interventions, Rt estimates remained close to 1 in both statewide and district-level analyses, with wide, overlapping 95% CrIs observed throughout (Figure 3). Percentage changes in policy Rt were also estimated for the state and its eight health districts (Figure 4; Table S3). Median estimates varied in both direction and magnitude across policies and districts. However, for all NPIs, the 95% CrIs crossed zero at both the state and district levels, indicating a high degree of uncertainty.

At the county level (Figure S6), median Rt values generally remained near 1, with wider credible intervals in counties with smaller populations. Variation across counties was slightly greater than across districts but remained modest overall. In the county-level change estimates (Figure S7), most median percentage changes were close to zero. Although a few smaller counties showed modest deviations, the credible intervals almost always overlapped with zero.

## 4. Discussion

This study assesses SARS-CoV-2 transmission dynamics in Maine from January 2020 through February 2023, focusing on regional variation and changes in the time-varying Rt following seven major policy-driven NPIs implemented in 2020. Four distinct waves of COVID-19 were identified; however, throughout the study period, Rt values generally fluctuated around 1 in both statewide and district-level analyses, including during periods of major policy interventions. No statistically significant changes in Rt were observed in association with any single policy. Estimated percentage changes in Rt during the defined NPI periods varied across districts, but, in all cases, the 95% CrIs overlapped zero, indicating substantial uncertainty. These findings align with prior studies that highlight the limitations of using Rt-based methods to infer policy impacts in low-incidence or rural settings, where underreporting, delayed testing, and sparse data can undermine the stability of transmission estimates [28,29].

Several factors may explain the absence of statistically significant Rt responses to the policy interventions examined. Underreporting and sparse data, particularly in rural or low-incidence areas, likely introduced noise into Rt estimates [37]. Inconsistent testing capacity and delayed reporting can mask true shifts in transmission, reducing the power to detect intervention effects [37]. Moreover, the effectiveness of policy measures depends not only on their timing or scope, but also on population adherence. Variation in compliance across districts, shaped by cultural norms, socioeconomic status, or perceived risk, may have diluted the observable impact of otherwise well-designed policies [38]. Even identical policies implemented at different times or in different settings may yield different outcomes due to behavioral and contextual factors. Future analyses could consider combining neighboring districts or isolating more urbanized regions to explore whether patterns become clearer in more homogeneous or data-rich settings [39,40].

Despite Maine’s early implementation of containment measures and relatively low case counts in 2020, local heterogeneity in transmission was evident. More densely populated districts, such as York (District 1) and Cumberland (District 2) (Table S4 and Figure S7), showed greater variation in Rt, although wide CrIs persisted across all districts. This spatial heterogeneity might reflect underlying differences in demographic composition, healthcare access, population density, and public adherence to interventions. These are well-established socioecological risk factors that have been shown to influence pandemic dynamics in other rural U.S. states [28].

Importantly, the inability to detect statistically significant policy effects in this analysis does not imply that NPIs were ineffective. Rather, it reflects both the methodological limitations of attributing changes in transmission to specific interventions, particularly in data-limited settings, and the broader, evolving context of the pandemic response. In the early stages of the pandemic, public health efforts focused on containing SARS-CoV-2 transmission. However, as it became increasingly clear that sustained suppression through prolonged restrictions was not feasible, many policies shifted toward mitigation strategies aimed at curbing transmission while minimizing socioeconomic disruption. These challenges underscore the importance of incorporating surveillance strategies, such as wastewater-based epidemiology and mobility data, to inform public health decision-making, especially in rural or low-incidence settings [19,41]. Future work may incorporate sociodemographic and behavioral data to better contextualize local responses and identify subpopulations at increased risk. As rural states continue to face structural barriers in healthcare infrastructure and public health preparedness, refined tools for real-time monitoring and policy evaluation will be critical to mitigating health disparities during future public health emergencies.

### Limitations

This study has several limitations. First, negative or zero case counts were addressed through imputation using local averaging. While this approach corrected reporting anomalies, the resulting values may not fully reflect actual case counts. Second, the underreporting of asymptomatic or mildly symptomatic infections introduced uncertainty in estimating the true scale of transmission. A Poisson-distributed multiplier of 4 was applied to estimate total infections from reported cases. Although noise was modeled using a Poisson distribution, the fixed mean value of the multiplier did not account for regional or temporal variation in testing coverage or case detection rates. Infection multipliers that vary by time and location were not available for Maine during the study period, which may have affected the accuracy of the estimated infection counts. Third, the *EpiEstim* package assumes a constant serial interval distribution, yet serial intervals can vary due to behavioral changes and public health responses. This simplification may have introduced bias, particularly if such responses lead to a reduction in the serial interval [42]. Fourth, the deconvolution method used to infer infection dates applies spline-based smoothing, which may have dampened short-term fluctuations in case counts caused by sudden shifts in behavior or policy. As a result, Rt estimates may have lagged or underestimated abrupt changes in transmission dynamics. Fifth, changes in Rt were analyzed across non-overlapping time windows defined by the policy implementation dates. While this method provided a structured basis for temporal comparison, the selected periods may not have aligned precisely with actual shifts in COVID-19 transmission or public behavior. The assumption that transmission remained stable within each window may have oversimplified real-world variability, particularly in the absence of real-time data on public adherence to NPIs. Sixth, counties were grouped into public health districts based on the state’s administrative structure. However, this spatial aggregation may have obscured important intra-district differences in transmission, healthcare access, or behavioral responses, limiting the geographic resolution of the analysis. This limitation is addressed in our sensitivity analysis of county-level data. Seventh, our study did not analyze mobility, adherence, or vaccination uptake data. Other studies have used human mobility data (e.g., Google COVID-19 Community Mobility Reports) [24,43,44,45] and aggregate data on government policy, adherence, and vaccine indicators (e.g., Oxford COVID-19 Government Response Tracker) [46], which capture population behavior and policy compliance more directly, to explain the effects of NPIs and vaccination in COVID-19 pandemic responses [47,48]. We acknowledge that their absence in our analysis left a gap in our understanding of the policy interventions’ impact, or the lack thereof, while we note that such additional analyses would be beyond the scope of this study. Finally, given the ecological study design, ecological fallacies are possible: associations observed at the aggregate level may not imply causal relationships at the individual level.

## 5. Conclusions

This study examined temporal and regional patterns in SARS-CoV-2 transmission across Maine using Rt estimates from January 2020 to February 2023. While no consistent or statistically significant changes in Rt were observed in association with major policy interventions implemented between April and November 2020, the analysis systematically evaluated transmission trends in a largely rural state. These findings serve as a reference point for understanding how transmission evolved over time and may inform future comparative analyses of public health responses in similar settings.

## Figures and Tables

**Table 1:** Major policy measures enacted in Maine in response to the COVID-19 pandemic, April–November 2020.

Label	Date	Policy Measure	Details
A	2 April 2020	Stay Healthy at Home Directive	Governor Mills issued a series of substantial new mandates to protect public health and safety in the face of COVID-19, including a *Stay Healthy at Home* directive that required people living in Maine to stay at home at all times, except for essential jobs or essential personal reasons such as obtaining food, medicine, health care, or other necessary purposes [30].
B	1 May 2020	Re-opening (Stage 1)	Governor Mills issued a new *Stay Safer at Home* Executive Order. The order continued to direct Maine residents to stay at home, with established exceptions for permitted activities such as occasional grocery shopping or exercising. It also allowed residents to visit businesses or participate in activities deemed safe to open under Stage 1 of the reopening plan presented on 28 April. These included barber shops and hair salons, auto dealerships, and drive-in, stay-in-your-vehicle religious services that followed COVID-19 Prevention Checklists. The order was effective immediately and extended through 31 May 2020, subject to change [31].
C	31 May 2020	Gradual Easing of Restrictions	Governor Mills signed an Executive Order allowing for the gradual easing of restrictions implemented under previous orders as the state continued to reopen under the *Restarting Maine’s Economy* plan. As of this date, Maine had reopened its economy on par with, or to a greater extent than, most other New England states [32].
D	8 July 2020	Face Covering Mandate (Certain Businesses)	Governor Mills issued an Executive Order requiring large retail businesses, restaurants, outdoor bars, tasting rooms, and lodging establishments in Maine’s more populous cities and coastal counties to enforce the state’s face covering requirement. Governor Mills also extended the State of Civil Emergency for thirty days through 6 August 2020 [33].
E	8 September 2020	School Re-opening	Students in Maine returned to in-person classes for the first time since schools were closed due to the pandemic [34].
F	13 October 2020	Re-opening (Stage 4)	The Mills Administration announced that Maine would move into Stage 4 of the *Plan to Restart Maine’s Economy* beginning Tuesday, 13 October 2020. Stage 4 increased limits on indoor seating to 50% of permitted occupancy, or 100 people (whichever was less) and maintained critical public health measures outlined in COVID-19 Prevention Checklists, such as enhanced cleaning practices and physical distancing. The state’s face covering mandate was further strengthened, requiring a broader range of entities, such as private schools and municipal buildings, to ensure that employees and individuals in their facilities adhered to this requirement. The order also expanded enforcement statewide, rather than limiting it to coastal counties and more populous cities [35].
G	4 November 2020	Face Covering Mandate (All Public Settings)	Governor Mills announced an Executive Order requiring Maine residents to wear face coverings in public settings, regardless of the ability to maintain physical distance [36].

## Data Availability

The COVID-19 data analyzed in this project is publicly available at the COVID-19 Data Repository by the Center for Systems Science and Engineering (CSSE) at Johns Hopkins University. The original contributions presented in this study are included in the article/Supplementary Material. Further inquiries can be directed to the corresponding author.

## Supplementary Materials

The following supporting information can be downloaded at: https://www.mdpi.com/article/10.3390/pathogens14090893/s1, File S1 includes a chronological list of COVID-19 policy measures enacted in Maine; tables of best-fitting degrees of freedom, percentage changes in Rt by policy period, and population data; and figures showing multiple Rt sensitivity analyses using alternative model parameters and serial interval distributions, as well as statewide and county-level changes in Rt following major policy interventions. File S2 (an RMarkdown file) includes the code used for data analysis and visualization; and File S3 (an R file) contains the custom R functions used in the analysis.

## Abbreviations

The following abbreviations are used in this manuscript:

CARES	Coronavirus Aid, Relief, and Economic Security
CDC	Centers for Disease Control and Prevention
COVID-19	Coronavirus Disease 2019
CSSE	Center for Systems Science and Engineering
CrI	Credible Interval
DACF	Department of Agriculture, Conservation, and Forestry
DHHS	Department of Health and Human Services
DOF	Degree of Freedom
MEMA	Maine Emergency Management Agency
NPI	Non-Pharmaceutical Intervention
OPTIONS	Overdose Prevention Through Intensive Outreach, Naloxone, and Safety
PPE	Personal Protective Equipment
Rt	Time-Varying Reproduction Number
SARS-CoV-2	Severe Acute Respiratory Syndrome Coronavirus 2
SD	Standard Deviation
VRBO	Vacation Rentals by Owner
