# Supplementary material for: COVID-19 Transmission Potential and Non-Pharmaceutical Interventions in Maine During the COVID-19 Pandemic
Source: Pathogens. 2025 Sep 5;14(9):893. doi: 10.3390/pathogens14090893 (PMC12472373; doi:10.3390/pathogens14090893)
Supplement: Supplementary file 1 [file pathogens-14-00893-s001.zip › pathogens-3756543-supplementary.pdf]

pathogens

MDPI

File S1 for COVID-19 Transmission Potential and Non-Pharmaceutical Interventions in Maine During the COVID-19 Pandemic

Table S1. Chronological list of policy measures enacted in Maine in response to the COVID-19 pandemic, March–December 2020.

Date	Policy Measure
March 2, 2020	Governor Mills convened a Coronavirus Response Team headed by Maine Center for Disease Control and Prevention (CDC) Director Dr. Nirav Shah and consisting of commissioners of the Departments of Health and Human Services; Defense, Veterans and Emergency Management; Public Safety; Education; Corrections; Labor; Transportation; and Administrative and Financial Affairs [1].
March 12, 2020	Governor Mills: 1) proclaimed an insurance emergency to improve access to care and required private health insurance plans to cover costs related to coronavirus testing; 2) suspended all non-essential out-of-state work travel by State employees; and 3) recommended, on the advice of the Maine CDC, that non-essential large indoor gatherings of 250 attendees or more be postponed in order to delay a potential coronavirus outbreak and substantially reduce its spread [2].
March 15, 2020	Governor Mills: 1) requested that the Small Business Administration provide economic support loans to Maine small businesses in order to help them overcome any temporary loss of revenue (request approved March 16; Maine was one of the first states nationwide to receive approval); and 2) submitted emergency legislation that temporarily revised eligibility for unemployment insurance to cover individuals whose employment had been impacted by COVID-19 and waived the one-week waiting period [3].
March 15, 2020	Governor Mills declared a Civil State of Emergency and recommended: 1) ending classroom instruction in all public schools as soon as reasonably practical; 2) postponing all non-urgent medical procedures, elective surgeries, and appointments at hospitals and health care providers across the state until further notice; 3) restricting visitors and all non-essential health care personnel to long-term care facilities except for certain compassionate care situations such as end-of-life care until further notice; and 4) postponing all events with 50 or more people, and all gatherings of more than 10 that included individuals at higher risk for severe illness, such as seniors, until further notice [4].
March 16, 2020	Governor Mills called for the statewide cancellation of Saint Patrick's Day events to prevent the gathering of large crowds and further encouraged social distancing measures [5].
March 16, 2020	Governor Mills reached a bipartisan agreement with legislative leaders on a revised supplemental budget proposal that protected the health and well-being of Maine people [6].
March 17, 2020	Governor Mills issued a Declaration of Abnormal Market Disruption. The declaration, drafted in close consultation with Attorney General Aaron M. Frey, prohibited certain necessities from being sold at unconscionable prices [7].
March 17, 2020	Governor Mills announced a package of emergency measures to respond to COVID-19, which were approved unanimously by the Legislature and signed by the Governor. The measures granted Governor Mills access to at least $11 million in State funding to respond to COVID-19, expanded authorities of State and local officials to allow them greater flexibility to respond to the virus, and provided support to Maine workers impacted by the virus [8].
March 18, 2020	Governor Mills issued an Executive Order mandating that all restaurants and bars statewide close to dine-in customers effective March 18, 2020, at 6:00 p.m. for a period of 14 days until midnight, March 31, 2020. Take-out, delivery, and drive-through options could continue. In her order, the Governor also prohibited all gatherings of more than 10 people until further notice, mandating the latest U.S. CDC guidance on gatherings. In addition, Governor Mills strongly urged non-essential public-facing businesses, such as gyms, hair salons, theatres, casinos, and shopping malls, to close their doors for the next two weeks to minimize public gatherings [9].
March 19, 2020	Governor Mills pressed the Federal government to provide more Personal Protective Equipment (PPE) and testing supplies to the State of Maine. In a letter to Vice President Mike Pence and U.S. Secretary of Health and Human Services Alex Azar, Governor Mills requested that the Federal government expedite

	the release of PPE from the Strategic National Stockpile and pushed for "a steady and reliable supply" of testing materials as the outbreak intensified [10].
March 19, 2020	Governor Mills convened a conference call with Maine's Federal Delegation to discuss the State's response to COVID-19 and the Congressional Delegation's work in Congress to support the State's efforts [11].
March 19, 2020	Governor Mills signed an Executive Order directing all Maine schools to continue paying non-salaried employees such as education technicians, food service employees, custodians, and other hourly-paid school employees for the duration of the school year [12].
March 20, 2020	Governor Mills suspended the inland waters fishing license requirement and opened waters to inland fishing until April 30 to encourage Maine people to enjoy the outdoors as they confronted the challenges associated with COVID-19 [13].
March 20, 2020	Governor Mills issued an Executive Order suspending provisions of certain health care professional licensing statutes and rules to facilitate the treatment and containment of COVID-19. The order authorized, among other measures, physicians, physician assistants, and nurses who were licensed in good standing in another state to be issued an emergency Maine license valid during the state of emergency [14].
March 23, 2020	Governor Mills issued an Executive Order extending compliance dates and authorizing the waiver of fees under certain motor vehicle laws until 30 days after the end of the emergency [15].
March 24, 2020	Governor Mills ordered all non-essential businesses and operations in Maine to close their physical locations that were public facing, meaning those that allowed customer, vendor, or other in-person contact. Governor Mills also strongly urged all large, essential, public-facing businesses to immediately employ strategies to reduce congestion in their stores, including limiting the number of customers in the store at any one time and enhancing curbside pick-up and delivery services [16].
March 25, 2020	The Mills Administration, through the Maine Emergency Management Agency, signed agreements with the University of Maine System and the Maine Community College System to coordinate requests for assistance and the deployment of resources to assist in Maine's response to the COVID-19 public health emergency [17].
March 25, 2020	Governor Mills signed an Executive Order to bolster the health care workforce and facilitate the expansion of telehealth by allowing licensed physicians, physician assistants, and nurses greater flexibility to contribute to Maine's response during the civil state of emergency. To implement the Governor's initiative, Superintendent of Insurance Eric Cioppa also signed an order that required insurance companies to provide coverage for clinically appropriate services delivered by telephone, as well as via more commonly used apps such as FaceTime, WhatsApp, and Skype, as long as they were private [18].
March 26, 2020	The Department of Health and Human Services began providing additional payments through MaineCare to support nursing facilities' efforts related to infection control and visitor screening to protect workers and the residents in their care [19].
March 26, 2020	Governor Mills and Commissioner of the Department of Administrative and Financial Services, Kirsten Figueroa, extended the deadline for Maine income tax payments from April 15, 2020, to July 15, 2020 [20].
March 26, 2020	The Department of Agriculture, Conservation and Forestry's Bureau of Parks and Lands closed the following coastal state parks until April 8: Reid State Park, Popham Beach State Park, Fort Popham, Fort Baldwin, Kettle Cove State Park, Two Lights State Park, Crescent Beach State Park, Scarborough Beach State Park, Ferry Beach State Park, and Mackworth Island [21].
March 27, 2020	In response to the extraordinary generosity of Maine people, businesses, and organizations looking to help during those challenging times, Governor Mills launched a new online resource, coronavirus.maine.gov/mainehelps, to outline ways in which Maine people could support efforts to combat the COVID-19 pandemic [22].
March 27, 2020	Governor Mills directed Maine Department of Inland Fisheries and Wildlife Commissioner Judy Camuso to allow boaters to operate without a current 2020 registration and milfoil sticker until April 30, 2020. Boat registrations normally expired at the end of each calendar year [23].
March 27, 2020	Governor Mills signed an Executive Order allowing Maine Quality Centers to swiftly address the changing demands in Maine's job market due to the widespread economic disruption caused by COVID-19 [24].

March 30, 2020	Governor Mills and Commissioner of the Department of Health and Human Services (DHHS), Jeanne Lambrew, announced that the Administration accelerated pay increases for personal care workers and expanded access to meals for older Mainers who were homebound because of COVID-19 [25].
April 1, 2020	Governor Mills submitted a request through the Federal Emergency Management Agency for a Presidential Major Disaster Declaration in light of the significant impact of COVID-19 on the State of Maine. If Governor Mills' request for assistance were approved, Maine people would have access to additional resources that supported child care, behavioral health, and legal services, among others, needed in response to the pandemic [26].
April 1, 2020	Governor Mills issued an Executive Order extending the State's property tax exemption deadline [27].
April 2, 2020	Governor Mills issued a series of substantial new mandates to protect public health and safety in the face of COVID-19, including a Stay Healthy at Home directive that required people living in Maine to stay at home at all times unless for an essential job or an essential personal reason, such as obtaining food, medicine, health care, or other necessary purposes [28].
April 3, 2020	Governor Mills issued an Executive Order mandating that travelers arriving in Maine, regardless of their state of residency, self-quarantine for 14 days to mitigate the spread of COVID-19. Additionally, the Order instructed visitors not to travel to Maine if they were displaying symptoms of COVID-19 and advised them not to travel to Maine if they were traveling from cities or regions identified as COVID-19 hot spots. To deter travel, the Order also suspended lodging operations, including hotels, motels, bed and breakfasts, inns, and short-term rentals such as those available through Vacation Rentals By Owner (VRBO), Airbnb, recreational vehicle parks and campgrounds, and all public and private camping facilities, as well as online reservations effective April 5, 2020, at 12:00 p.m. [29].
April 4, 2020	Governor Mills announced that the President had approved her request for a major disaster declaration for the State of Maine, unlocking critical federal assistance for state agencies and municipalities in Maine [30].
April 7, 2020	Governor Mills signed an Executive Order to take additional steps to bolster the health care workforce, expand telehealth services, and temporarily alleviate certain licensing requirements, building upon the Governor's March 20 Executive Order [31].
April 7, 2020	Governor Mills directed the Maine National Guard and the Maine Emergency Management Agency (MEMA) to work with Maine's health care systems to open two alternative care sites in Portland and Bangor as part of the State's preparations to bolster Maine's health system capacity in the face of COVID-19 [32].
April 8, 2020	The Maine Department of Education delivered 500 Wi-Fi-enabled Samsung Tab A's with 12 months of Verizon service to Piscataquis County schools, in an effort that helped students in the area with their schoolwork while they were engaged in remote learning during the COVID-19 pandemic [33].
April 8, 2020	To reduce in-person contact and promote physical distancing in response to COVID-19, Governor Mills signed an Executive Order allowing remote notarization [34].
April 10, 2020	Governor Mills signed an Executive Order moving Maine's primary election from Tuesday, June 9, 2020, to Tuesday, July 14, 2020. The Order, which was effective immediately, also allowed applications for absentee ballots to be made in writing or in person, without specifying a reason, up to and including the day of the election. It also extended the deadline for qualifying contributions under the Maine Clean Election Act to May 19, 2020 [35].
April 11, 2020	Governor Mills directed DHHS Commissioner Jeanne Lambrew to take a series of actions to ensure that Maine people affected by Substance Use Disorder could access critical treatment and resources in the face of COVID-19, including facilitating access to medication and counseling, promoting harm reduction strategies, and accelerating financial relief for providers [36].
April 13, 2020	The Maine Department of Economic and Community Development launched a program to identify startups and early-stage technology companies with products to aid the COVID-19 response [37].
April 14, 2020	Governor Mills signed a proclamation extending Maine's state of civil emergency for another thirty days through May 15, 2020 [38].
April 14, 2020	The Maine Department of Labor announced that Mainers receiving unemployment benefits would begin to see the additional weekly $600, created by the federal Coronavirus Aid, Relief, and Economic Security (CARES) Act, by early the following week, and that the unemployment phone line would be open from

	8 a.m. to 3 p.m. The amount paid in unemployment insurance benefits to Maine people since March 16 was over $46 million [39].
April 15, 2020	As the State continued to respond to COVID-19, Governor Mills signed a proclamation extending Maine's state of civil emergency for another thirty days through May 15, 2020. The original state of civil emergency had been scheduled to expire on April 15, 2020 [40].
April 16, 2020	Governor Mills signed an Executive Order to limit evictions during the COVID-19 state of emergency, strengthen penalties for landlords who tried to evict tenants by unlawful means, and extend timeframes for eviction proceedings if courts reopened before the state of emergency ended [41].
April 16, 2020	Governor Mills and MaineHousing announced a $5 million COVID-19 Rent Relief Program for households that met certain income requirements, and the Governor urged all financial institutions to help Maine homeowners and small businesses experiencing financial hardship from COVID-19 to stay in their homes and storefronts [42].
April 21, 2020	The Maine Department of Health and Human Services launched the FrontLine WarmLine to serve health care professionals, as well as emergency medical services personnel, law enforcement, and others who were directly responding to the pandemic in Maine. The phone line was staffed by volunteer professionals activated through Maine Responds, including licensed psychiatrists, psychologists, therapists, social workers, and nurse practitioners, who could help callers deal with anxiety, irritability, stress, poor sleep, grief, or worry and, if needed, connect them with additional supports. The FrontLine WarmLine was available to clinicians and first responders from 8 a.m. to 8 p.m., 7 days a week [43].
April 21, 2020	In order to provide more opportunities for both youth and adult turkey hunters and abide by current executive orders, Commissioner Judy Camuso, with the support of Governor Mills, opened the turkey season early, providing hunters with additional days to hunt that spring season, and suspended the requirement to register harvested turkeys [44].
April 22, 2020	Governor Mills and Maine DHHS Commissioner Jeanne Lambrew announced that nearly $11 million in federal funds would support access to child care for Maine's essential workers, including health care professionals and first responders, and offer relief for child care providers in response to the COVID-19 pandemic [45].
April 23, 2020	Governor Mills outlined her vision for a gradual and safe reopening of Maine's economy amid the COVID-19 pandemic, emphasizing four principles that would guide the State's decision-making process and inviting Maine people to offer their ideas through a new portal launched by the Department of Economic and Community Development [46].
April 27, 2020	Governor Mills announced that the Consensus Economic Forecasting Commission and the Revenue Forecasting Committee would convene off-cycle to examine the economic ramifications of COVID-19 on State government's revenues [47].
April 28, 2020	Governor Mills presented her Administration's plan to gradually and safely restart Maine's economy. The plan, which came as the State appeared to be successfully flattening the curve, established four gradual stages of reopening, the first of which began May 1. The Governor also announced she would extend the State's stay-at-home order in the form of a new "Stay Safer at Home" Executive Order [48].
May 1, 2020	Governor Mills issued a new Stay Safer at Home Executive Order. The new order continued to have Maine people stay at home with the same established exceptions for permitted activities, such as occasional grocery shopping or exercising. However, it also allowed Maine people to visit businesses or participate in activities deemed safe to open under Stage 1 of the reopening plan presented on April 28. These included barber shops and hair salons, auto dealerships, and drive-in stay-in-your-vehicle religious services that followed COVID-19 Prevention Checklists. The Order was effective immediately and extended through May 31, 2020, subject to change [49].
May 6, 2020	Governor Mills signed an Executive Order convening an Economic Recovery Committee charged with developing recommendations to mitigate the damage to Maine's economy caused by the ongoing COVID-19 pandemic and to jumpstart a long-term economic recovery for Maine people, businesses, and organizations. The thirty-seven-member Committee included representatives of small businesses, nonprofits, financial institutions, unions, municipalities, tribal and immigrant communities, hospitality and tourism industries, and educational institutions, as well as a bipartisan delegation of legislative designees [50].

May 7, 2020	Governor Mills announced that her Administration had partnered with Maine-based IDEXX Laboratories, Inc. to purchase enough of the company's recently authorized COVID-19 testing kits to more than triple the State's testing capacity. The breakthrough would soon allow anyone in Maine suspected of having COVID-19 to receive a test [51].
May 8, 2020	Governor Mills announced a rural reopening plan aimed at reopening certain additional businesses in rural Maine with added health and safety measures in the following two weeks. Under the plan, retail stores and restaurants would be permitted to open to in-store and some dine-in service, respectively—with enhanced safety precautions—in counties where community transmission was not present. Those counties were Aroostook, Piscataquis, Washington, Hancock, Somerset, Franklin, Oxford, Kennebec, Waldo, Knox, Lincoln, and Sagadahoc [52].
May 12, 2020	The Mills Administration launched a statewide campaign to promote awareness of affordable health insurance options, particularly for people whose employment or income had been affected by the COVID-19 pandemic [53].
May 13, 2020	Governor Mills signed a proclamation extending Maine's state of civil emergency for thirty days through June 11, 2020 [54].
May 14, 2020	The Mills Administration announced that, effective immediately, Maine lodging providers could begin accepting future reservations for stays with an arrival date of June 1 and beyond for Maine residents and for non-residents who complied with the State's 14-day quarantine requirement [55].
May 15, 2020	Governor Mills and Maine Education Commissioner Pender Makin announced that the Maine Department of Education had secured internet access and devices to facilitate at-home learning for 100 percent of Maine school children for whom there was a reported need [56].
May 18, 2020	To further protect the health and safety of essential employees in Maine, including members of the media, MEMA provided 1,900 cloth face coverings to the Maine Association of Broadcasters for distribution to the state's broadcast media outlets [57].
May 19, 2020	The Mills Administration announced the following updates to its plan to restart Maine's economy: 1) Maine residents could enjoy private campgrounds beginning Memorial Day weekend; and 2) the Administration was delaying the full reopening of gyms, fitness centers, and nail salons in light of emerging research and experiences in other states of COVID-19 transmission related to these establishments [58].
May 21, 2020	Governor Mills announced that the State of Maine had received $52.7 million in federal grant funding from the U.S. CDC to bolster epidemiological and laboratory capacity to respond to infectious diseases, particularly COVID-19 [59].
May 26, 2020	Governor Mills announced that the Maine DHHS was significantly expanding contact tracing by increasing the number of skilled staff and volunteers, harnessing innovative technology, and securing social services to help people with COVID-19 maintain self-isolation [60].
May 27, 2020	The Mills Administration announced that it was postponing the full reopening of restaurants for dine-in services in York, Cumberland, and Androscoggin counties. Restaurants in these counties had been tentatively scheduled to reopen to dine-in services on June 1 (Stage 2) but were instead restricted to reopening to outside dining service only beginning on that date, in addition to continuing to provide take-away and delivery services [61].
May 31, 2020	Governor Mills signed an Executive Order that allowed for the gradual easing of restrictions implemented under previous Executive Orders as the state continued to reopen under the Restarting Maine's Economy plan. At that time, Maine had reopened its economy on par with or to a greater extent than most other New England states [62].
May 30, 2020	Governor Mills directed the Maine Department of Corrections to offer to purchase perishable and non-perishable food from restaurants in York, Cumberland, and Androscoggin counties that were unable to open to indoor dining as a result of measures to protect public health and mitigate the spread of COVID-19 [63].
June 4, 2020	Governor Mills and MaineHousing announced an extension of the COVID-19 Rent Relief Program [64].
June 4, 2020	The Mills Administration announced additional business reopenings under the Governor's rural reopening plan. Under the update, in 13 counties, tasting rooms and bars could open for outside service, and gyms and fitness centers, nail salons, and tattoo and piercing parlors could open with added health and

	safety precautions beginning on Friday, June 12. These establishments were allowed to reopen everywhere in all counties except for York, Cumberland, and Androscoggin counties [65].
June 8, 2020	The Mills Administration announced that it would quadruple COVID-19 testing capacity at the State lab, develop testing sites throughout Maine, and allow more people in Maine with elevated risk of exposure to get tested for the virus [66].
June 8, 2020	The Mills Administration unveiled an alternative to the State's 14-day quarantine requirement for people entering Maine. The multilayered plan, called Keep Maine Healthy, aimed to protect the health of Maine people and visitors while allowing the opportunity for people to visit Maine and support Maine small businesses during the summer months [67].
June 10, 2020	As the State continued to respond to COVID-19, Governor Mills extended the State of Civil Emergency for thirty days through July 10, 2020 [68].
June 12, 2020	The Mills Administration announced that it had accelerated the first day that lodging establishments in Maine could begin serving out-of-state visitors who met either the 14-day quarantine requirement or the State's new testing alternative [69].
June 12, 2020	The Maine DHHS announced that 525 essential workers had received financial help for child care and more than 1,600 child care providers had received stipends as part of the award of nearly $11 million in federal CARES Act funding to Maine. Additional grants were scheduled to be awarded to child care providers through mid-July [70].
June 15, 2020	The Mills Administration announced that indoor dining in Androscoggin, Cumberland, and York counties could voluntarily resume on Wednesday, June 17, 2020, with added health and safety protocols outlined in the COVID-19 Prevention Checklist [71].
June 15, 2020	The Mills Administration released detailed guidance for Maine local governing bodies to hold gatherings, such as annual town meetings, school budget meetings, and elections, during the COVID-19 pandemic [72].
June 22, 2020	Governor Mills called for the United States Department of Agriculture to designate maple syrup as an eligible Coronavirus Food Assistance Program specialty crop [73].
June 22, 2020	The Mills Administration announced that it was postponing the reopening of indoor service at bars to protect public health given the higher risk of COVID-19 transmission in such settings [74].
June 24, 2020	The Mills Administration announced that it was dedicating $35 million of Maine's $1.25 billion in Coronavirus Relief Funding provided through the CARES Act to help local and tribal governments and other qualified entities cover costs incurred as a result of COVID-19 [75].
June 26, 2020	Governor Mills announced that her Administration had approved almost $9 million in grant awards to nearly 100 municipalities across the state under the Keep Maine Healthy Plan to support local COVID-19 public health, education, and prevention efforts [76].
July 1, 2020	The Mills Administration announced that it was exempting visitors from the States of Connecticut, New York, and New Jersey from the 14-day quarantine requirement or negative COVID-19 testing alternative, effective Friday, July 3, 2020 [77].
July 8, 2020	Governor Mills issued an Executive Order requiring large retail businesses, restaurants, outdoor bars, tasting rooms, and lodging establishments in Maine's more populous cities and coastal counties to enforce the State's face covering requirement. Governor Mills also extended the State of Civil Emergency for thirty days through August 6, 2020 [78].
July 14, 2020	The Mills Administration announced that seven health care organizations would collectively launch nearly 20 "swab and send" COVID-19 test collection sites that would send samples to the Maine State Lab for testing, strengthening access to safe and accessible testing for residents, tourists, seasonal workers, and other visitors to Maine [79].
July 15, 2020	Governor Mills announced that her Economic Recovery Committee had delivered to her its immediate recommendations to stabilize and support Maine's economy through the unprecedented disruption caused by the COVID-19 pandemic [80].
July 17, 2020	The Mills Administration announced that it would invest more than $8 million from the federal Coronavirus Relief Fund to further boost access to child care and support Maine's working families in response to the COVID-19 pandemic [81].

July 17, 2020	Governor Mills announced a series of steps her Administration was taking to assist and support school systems across Maine as they considered whether and how to return to in-classroom instruction that fall [82].
July 21, 2020	The Maine DHHS announced four additional COVID-19 "swab and send" testing sites, the award of $1 million in federal funding to rural hospitals to increase their labs' capacity to process COVID-19 tests, and a second round of grants to municipalities under the Keep Maine Healthy Plan to support local COVID-19 public health, education, and prevention efforts [83].
July 30, 2020	The Mills Administration announced an investment of $1 million from the Coronavirus Relief Fund to significantly and quickly expand services to help reduce the disproportionately large racial and ethnic disparities in COVID-19 in Maine [84].
July 30, 2020	Governor Mills announced that MaineHousing would double its rental assistance from $500 to $1,000 through the COVID-19 Rental Relief Program beginning Monday, August 3. The Governor also signed an Executive Order continuing expanded timeframe protections for renters in the eviction process [85].
August 5, 2020	Governor Mills extended the State of Civil Emergency for thirty days through September 3, 2020 [86].
August 11, 2020	The Mills Administration announced that it had approved an additional $4 million in awards to more than 80 municipalities and Tribal governments across the state under a second round of Keep Maine Healthy funding to support local COVID-19 public health, education, and prevention efforts [87].
August 20, 2020	The Maine DHHS and MaineHealth announced the launch of five new "swab and send' COVID-19 testing sites, further expanding access to reliable and timely testing for Maine people and visitors [88].
August 20, 2020	Governor Mills announced the Maine Economic Recovery Grant Program to support Maine businesses and nonprofits as they continued to grapple with economic hardships caused by COVID-19. The program, backed by $200 million in federal CARES Act Coronavirus Relief Funds, would provide financial relief for businesses and non-profit organizations that had incurred business disruptions as a result of the COVID-19 pandemic [89].
August 27, 2020	Governor Mills announced she had signed an Executive Order to protect the health and safety of Maine voters, poll workers, and election officials and to facilitate access to safe in-person and absentee voting in the November general election amid the ongoing COVID-19 pandemic [90].
September 2, 2020	Governor Mills extended the State of Civil Emergency for thirty days through October 1, 2020 [91].
September 17, 2020	Governor Mills signed a curtailment order to maintain budget stability amid a projected revenue shortfall caused by the COVID-19 pandemic. The Executive Order, which adopted recommendations from the Department of Administrative and Financial Services, curtailed allotments to the State's General Fund by $221,775,584 and to the Highway Fund by $23,000,822 [92].
September 21, 2020	Governor Mills launched a second phase of the Maine Economic Recovery Grant Program beginning Wednesday, September 23, 2020. Phase 2 made available approximately $95 million in remaining funds from Phase 1 and expanded access to the program by increasing the number of eligible businesses and non-profits [93].
September 22, 2020	The Maine DHHS broadened its Standing Order to include all individuals who thought they needed a COVID-19 test. This meant that participating sites could test anyone in Maine over the age of 12 months who felt they needed a test, even if they didn't have a primary care provider or a written order from a clinician [94].
September 23, 2020	Governor Mills dedicated an additional $164 million in CARES Act Coronavirus Relief Funds to support school systems as they continued to implement health and safety protocols to protect students, staff, and their families amid the COVID-19 pandemic [95].
September 23, 2020	The Mills Administration announced that, effective immediately, travelers from Massachusetts were exempt from the requirement to get a negative COVID-19 test or quarantine for 14 days [96].
September 30, 2020	Governor Mills extended the State of Civil Emergency for thirty days through October 29, 2020 [97].

October 13, 2020	The Mills Administration announced that Maine would move into Stage 4 of the Plan to Restart Maine's Economy beginning Tuesday, October 13, 2020. Stage 4 increased limits on indoor seating to 50 percent of permitted occupancy, or 100 people—whichever was less—and maintained the critical public health measures outlined in COVID-19 Prevention Checklists, such as enhanced cleaning practices and physical distancing. The Executive Order also further strengthened the State's face covering mandate by requiring that a broader set of entities, such as private schools and municipal buildings, ensure that employees and people in their buildings adhered to this critical health measure. The Order also expanded the scope of enforcement statewide, rather than just in Maine's coastal counties and more populous cities [98].
October 15, 2020	Governor Mills and Commissioner of Economic and Community Development Heather Johnson announced that the Mills Administration had begun distributing the first round of financial awards to Maine small businesses and non-profits through the Maine Economic Recovery Grant Program. The 2,329 awards, which included 2,072 to small businesses and 257 to non-profits, totaled $105 million and averaged just over $45,000 per award, with recipients spanning the entire state [99].
October 21, 2020	To combat the disturbing rise in fatal drug overdoses exacerbated by the COVID-19 pandemic, Governor Mills announced a new "OPTIONS" (Overdose Prevention Through Intensive Outreach, Naloxone and Safety) initiative. Under the initiative, mobile response teams in every Maine county would engage with communities that had high rates of drug overdoses to promote drug prevention and harm reduction strategies, connect people directly to recovery services and treatment, and distribute naloxone [100].
October 29, 2020	Governor Mills extended the State of Civil Emergency for thirty days through November 27, 2020 [101].
October 29, 2020	The Maine DHHS announced its strategy to distribute an allocation of rapid COVID-19 antigen tests to ensure access for all Maine residents, especially those at greater risk of exposure to the virus. DHHS partnered with Walgreens to distribute the bulk of the tests, approximately 300,000, to 65 pharmacy locations from Kittery to Madawaska. This testing was available to the public at no charge as a drive-through service in November [102].
November 1, 2020	As the COVID-19 virus spread rapidly across the country, the Mills Administration announced actions to prevent and mitigate the spread of the virus, taking into consideration the need to keep businesses open and schools accessible to students. The Administration extended the "Keep Maine Healthy" program through December to promote local prevention efforts. It also returned to lower indoor gathering limits, postponed bar and tasting room reopenings, and removed New York, New Jersey, and Connecticut from exempt status under the State's travel advisory [103].
November 4, 2020	Governor Mills announced an Executive Order requiring Maine people to wear face coverings in public settings, regardless of the ability to maintain physical distance [104].
November 12, 2020	In response to recent coronavirus outbreaks associated with interstate youth hockey activities, New Jersey Governor Phil Murphy, Maine Governor Mills, Rhode Island Governor Gina Raimondo, New Hampshire Governor Chris Sununu, Vermont Governor Phil Scott, Connecticut Governor Ned Lamont, and Massachusetts Governor Charlie Baker announced they would support a regional approach to interstate competitions [105].
November 13, 2020	The Mills Administration announced that, effective Monday, November 16, 2020, Massachusetts was no longer exempt from Maine's quarantine or negative test requirement [106].
November 19, 2020	Beginning Friday, November 20, 2020, through Sunday, December 6, 2020, all outdoor and indoor amusement venues, movie theaters, performing arts venues, casinos, and businesses that provided seated food and drink service—including social clubs, restaurants, and bars and tasting rooms that were open for outdoor service—were required to close for that period. In addition, 52 Walgreens pharmacies began offering free drive-through rapid COVID-19 antigen testing on Friday to people in Maine experiencing symptoms of the virus. Three more Walgreens sites in southern Maine began offering antigen testing the following week, bringing the total to 65 Walgreens sites throughout the state [107].
November 20, 2020	Governor Mills announced that her Administration had awarded $5.6 million in CARES Act Coronavirus Relief Fund monies to fund the construction of permanent internet infrastructure that would bring high-speed broadband to more than 730 students across rural Maine [108].

November 20, 2020	Governor Mills and Commissioner of the Department of Economic and Community Development Heather Johnson announced that the Mills Administration had begun distributing the second round of financial awards to Maine small businesses and non-profits through the Maine Economic Recovery Grant Program. The 1,222 awards, which included 1,107 to businesses and 115 to non-profits, totaled $53.6 million and averaged just over $43,000 per award, with recipients spanning the entire state. In addition, the Maine Economic Recovery Grant provided $7.4 million in grants to 193 businesses and non-profits that were less than a year old [109].
November 20, 2020	To support Maine families struggling economically because of the ongoing coronavirus pandemic, Governor Mills announced she was dedicating an additional $6.2 million in federal Coronavirus Relief Funds to MaineHousing's COVID-19 Rental Relief Program. MaineHousing extended the program through December for renters who could not afford to pay their rent due to COVID-19 [110].
November 24, 2020	Governor Mills extended the State of Civil Emergency through December 23, 2020 [111].
November 25, 2020	The Mills Administration announced that it would dedicate more than half a million dollars in Coronavirus Relief Funds to extend home-delivered meals to older Mainers and caregivers, ensuring food and nutrition for Maine people staying home during the pandemic [112].
November 30, 2020	Governor Mills announced an economic recovery grant program to support Maine's tourism, hospitality, and retail small businesses. Backed by $40 million in federal CARES Act Coronavirus Relief Funds, the Tourism, Hospitality & Retail Recovery Grant Program was focused specifically on supporting Maine's service-sector small businesses—such as restaurants, bars, tasting rooms, lodging, and retail shops—which had been hard hit by the COVID-19 pandemic and faced additional challenges with the coming winter months [113].
November 30, 2020	Governor Mills and Health and Human Services Commissioner Jeanne Lambrew announced a grant program to support health care organizations that served residents with MaineCare and to sustain vital health services during the COVID-19 pandemic [114].
December 4, 2020	The Mills Administration announced it would extend the requirement for certain businesses statewide to close by 9:00 p.m. through Sunday, January 3, 2021 [115].
December 4, 2020	Governor Mills and Maine Department of Agriculture, Conservation and Forestry (DACF) Commissioner Amanda Beal announced that the Administration was committing $2 million in federal CARES Act Coronavirus Relief Funding to DACF's Food Security Network Reimbursement Program [116].
December 4, 2020	Governor Mills and Commissioner of Economic and Community Development Heather Johnson announced that the Administration was committing $10 million in federal CARES Act Coronavirus Relief Funding to a newly created Agriculture and Food Processing Infrastructure Reimbursement Program [117].
December 9, 2020	The Maine Department of Marine Resources, with support from Governor Mills, launched a branding and promotion initiative to celebrate Maine Seafood and help consumers find and enjoy it in the comfort and safety of their homes. With $1,000,000 of the $20 million CARES Act relief funds allocated by the National Oceanic and Atmospheric Administration for Maine's commercial fishing and seafood industry, the Maine Seafood branding and promotion initiative aimed to help this growing population of home-bound seafood enthusiasts discover the superior taste, quality, and variety of seafood from Maine [118].
December 9, 2020	Governor Mills and Commissioner of Labor Laura Fortman announced that the Administration would provide certain individuals who were unemployed due to COVID-19 with a one-time direct relief payment of $600 through the State's newly created Pandemic Relief Program [119].
December 11, 2020	Governor Mills and Commissioner for the Department of Administrative and Financial Services Kirsten Figueroa announced that the Administration had committed all of Maine's $1.25 billion in CARES Act Coronavirus Relief Funds [120].
December 11, 2020	With widespread community transmission and increased COVID-19 cases and hospitalizations in Maine, Governor Mills signed an Executive Order that simplified and strengthened the enforcement of the State's face covering requirement. Moving forward, owners and operators of all indoor public spaces—regardless of the type of entity or size—were not allowed to permit those who refused to wear a face

	covering to enter or remain in their venue. Previous Executive Orders had required enforcement in some but not all public settings [121].
December 22, 2020	Governor Mills extended the State of Civil Emergency through January 20, 2021 [122].
December 30, 2020	Amid an increase in Maine's COVID-19 positivity rate and a continued high rate of hospitalizations, the Mills Administration announced that it would extend the requirement for certain businesses statewide to close by 9:00 p.m. in order to limit activities that could contribute to the transmission of COVID-19 [123].
December 31, 2020	New Jersey Governor Phil Murphy, Maine Governor Mills, Rhode Island Governor Gina Raimondo, New Hampshire Governor Chris Sununu, Vermont Governor Phil Scott, Connecticut Governor Ned Lamont, and Massachusetts Governor Charlie Baker announced an extension of the suspension of interstate youth hockey competitions for public and private schools and youth hockey leagues through at least January 31, 2021 [124].

Table S2. Best-fitting degrees of freedom (DOF) for spline-based deconvolution models used to estimate infection time series for Maine statewide and across its eight public health districts. The best-fitting DOF was selected from a grid search over the range of 40–60 (in increments of 2) to balance model smoothness with fidelity to observed case count trends.

Public Health District	Best Degree of Freedom
Maine statewide	56
District 1	56
District 2	56
District 3	56
District 4	56
District 5	56
District 6	56
District 7	56
District 8	54

Table S3. Percentage changes in median Rt and corresponding 95% credible intervals (CrI) by policy period for Maine statewide and its eight public health districts.

Public Health District	Stay Healthy at Home Directive (%)	Re-opening (Stage 1) (%)	Gradual Easing of Restrictions (%)	Face Covering Mandate (Certain Businesses) (%)	School Re-opening (%)	Re-opening (Stage 4) (%)	Face Covering Mandate (All Public Settings) (%)
Maine statewide	-6.0 (-12.5, 2.2)	-3.6 (-12.1, 8.5)	-3.1 (-10.6, 13.9)	3.3 (-5.6, 9.4)	2.6 (-8.5, 11.7)	6.5 (-6.5, 17.8)	-3.4 (-10.9, 6.8)
District 1	-5.8 (-17.9, 8.2)	-2.4 (-15.7, 14.7)	-0.4 (-12.7, 20.2)	3.3 (-8.0, 15.0)	-2.6 (-15.8, 8.8)	10.1 (-5.1, 24.8)	-1.6 (-10.7, 10.9)
District 2	-5.9 (-14.6, 4.4)	-4.7 (-14.3, 8.4)	-5.1 (-14.6, 12.1)	1.5 (-8.8, 11.3)	9.4 (-4.6, 22.6)	6.1 (-9.1, 19.8)	-3.9 (-12.4, 8.0)
District 3	-4.6 (-16.9, 9.6)	-5.3 (-16.9, 9.5)	-4.1 (-14.9, 14.4)	4.2 (-7.0, 15.1)	1.6 (-11.9, 13.7)	8.8 (-6.5, 23.0)	-5.1 (-13.8, 6.4)
District 4	-7.8 (-19.2, 5.8)	-2.1 (-15.4, 15.0)	0.4 (-12.3, 21.7)	1.2 (-10.5, 13.8)	1.4 (-12.3, 14.0)	3.6 (-11.8, 18.1)	-1.9 (-12.0, 10.5)
District 5	-4.5 (-19.6, 13.2)	-1.6 (-18.0, 19.6)	0.3 (-15.2, 23.6)	3.2 (-10.1, 18.1)	0.2 (-13.6, 13.4)	4.7 (-10.3, 18.4)	-4.3 (-13.3, 7.2)
District 6	-4.8 (-21.5, 15.4)	-2.5 (-19.2, 19.1)	0.3 (-15.1, 23.5)	-0.6 (-13.5, 13.7)	2.7 (-12.2, 17.5)	9.7 (-7.0, 26.8)	-6.7 (-16.1, 5.8)
District 7	-3.9 (-23.0, 20.0)	-2.5 (-20.4, 20.7)	-1.2 (-17.0, 22.0)	-0.4 (-13.9, 14.6)	2.6 (-12.7, 18.0)	5.5 (-11.2, 23.1)	-5.6 (-16.3, 7.3)
District 8	-3.5 (-30.9, 35.9)	-3.0 (-26.1, 29.3)	-3.0 (-22.8, 25.4)	0.0 (-17.7, 21.5)	2.3 (-17.5, 25.1)	8.4 (-13.1, 34.9)	-1.4 (-15.7, 17.1)

Table S4. Total population and population density (number of people per square mile) for each public health district in Maine in 2020.

Public Health District	County	Population	Land Area (square miles)	Population Density by County (people/mi²)	Population Density by Public Health District (people/mi²)
District 1	York	206,074	991.2	207.90	207.90
District 2	Cumberland	294,520	836.2	352.21	352.21
District 3	Androscoggin	107,958	468.0	230.68	46.12
	Oxford	57,741	2,077.0	27.80	
	Franklin	29,933	1,697.0	17.64	
District 4	Knox	39,809	365.1	109.04	82.92
	Waldo	39,723	730.0	54.42	
	Sagadahoc	35,720	254.0	140.63	
	Lincoln	34,415	455.9	75.49	
District 5	Kennebec	122,158	867.5	140.82	36.05
	Somerset	50,573	3,924.3	12.89	
District 6	Penobscot	151,696	3,397.2	44.65	22.91
	Piscataquis	16,864	3,960.8	4.26	
District 7	Hancock	54,832	1,587.1	34.55	20.77
	Washington	31,378	2,562.7	12.24	
District 8	Aroostook	67,431	6,671.1	10.11	10.11

Figure S1. Sensitivity analysis of the 7-day sliding window Rt in Maine from January 22, 2020, to February 14, 2023. The *dof_grid* parameter was specified as a default vector of degrees of freedom ranging from 6 to 20 in increments of 2. The Rt was estimated using the Omicron-specific serial interval distribution, as in the main analysis, with a mean of 2.9 days and a standard deviation of 1.64 days. The prior distribution of Rt was specified with a mean of 2 and a standard deviation of 2. The median 7-day sliding window Rt is represented by the black line, with 95% CrI shaded in grey.

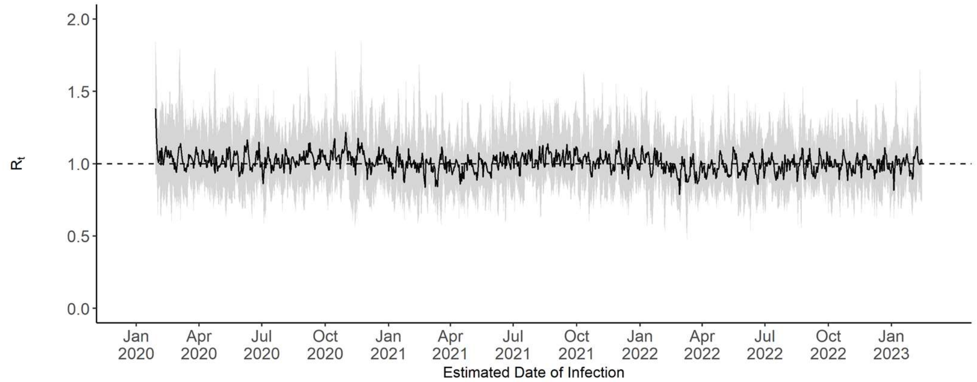

Figure S2. Sensitivity analysis of the 7-day sliding window Rt in Maine from January 22, 2020, to February 14, 2023. The *dof_grid* parameter was specified as a vector of degrees of freedom ranging from 40 to 60 in increments of 2. The Rt was estimated using the Omicron-specific serial interval distribution, as in the main analysis, with a mean of 2.9 days and a standard deviation of 1.64 days. The prior distribution of Rt was specified with a default mean of 5 and a default standard deviation of 5. The median 7-day sliding window Rt is represented by the black line, with 95% CrI shaded in grey.

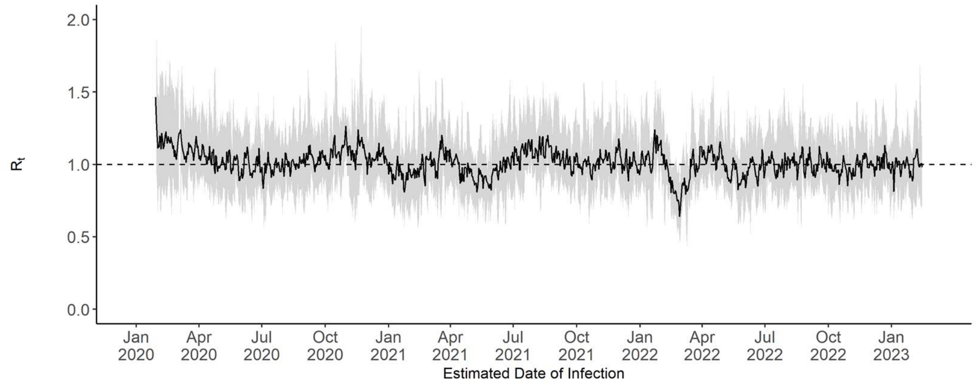

Figure S3. Sensitivity analysis of the 7-day sliding window Rt in Maine from January 22, 2020, to February 14, 2023. The *dof_grid* parameter was specified as a vector of degrees of freedom ranging from 40 to 60 in increments of 2. The Rt was estimated using an alternative serial interval distribution derived from early pandemic data, with a mean of 4.6 days and a standard deviation of 5.55 days. The prior distribution of Rt was specified with a mean of 2 and a standard deviation of 2. The median 7-day sliding window Rt is represented by the black line, with 95% CrI shaded in grey.

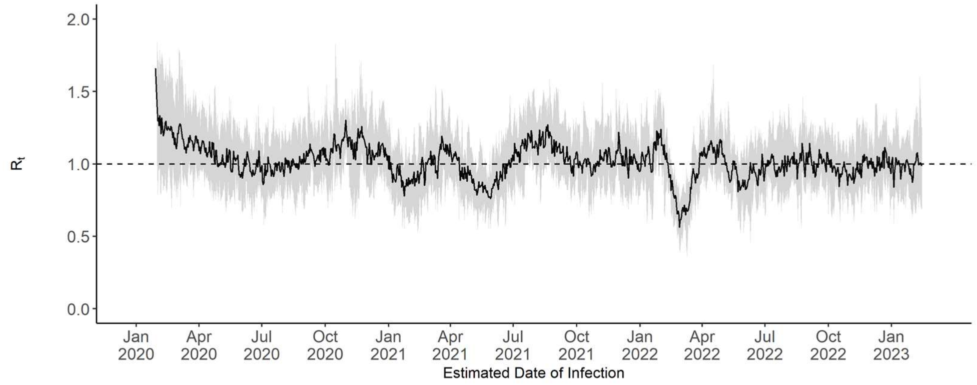

Figure S4. Sensitivity analysis of the 7-day sliding window Rt in Maine from January 22, 2020, to February 14, 2023. The *dof_grid* parameter was specified as a vector of degrees of freedom ranging from 40 to 60 in increments of 2. The Rt was estimated using an alternative serial interval distribution derived from early pandemic data, with a mean of 4.6 days and a standard deviation of 5.55 days. The prior distribution of Rt was specified with a default mean of 5 and a default standard deviation of 5. The median 7-day sliding window Rt is represented by the black line, with 95% CrI shaded in grey.

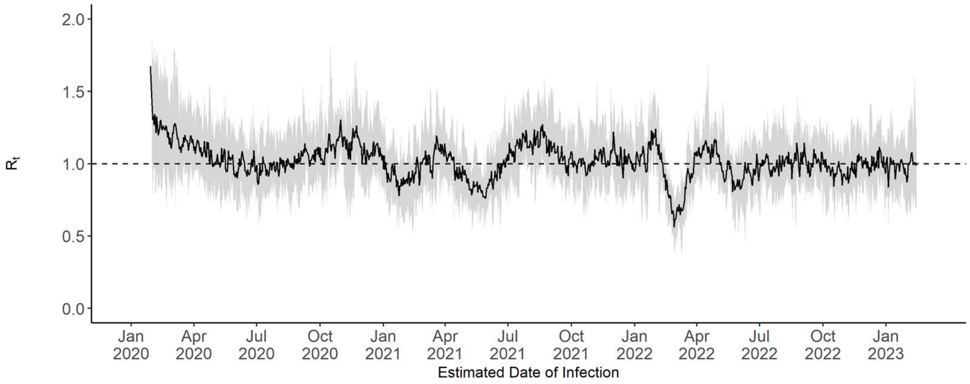

Figure S5. Sensitivity analysis of estimated infection counts and 7-day sliding window Rt with corresponding 95% CrI for Maine statewide from January 22, 2020, to February 14, 2023, using two infection multipliers (3.4 in blue, 4.7 in red). The upper panel shows the median estimated number of new infections with the corresponding 95% CrI. The lower panel shows the median 7-day sliding window Rt with the corresponding 95% CrI.

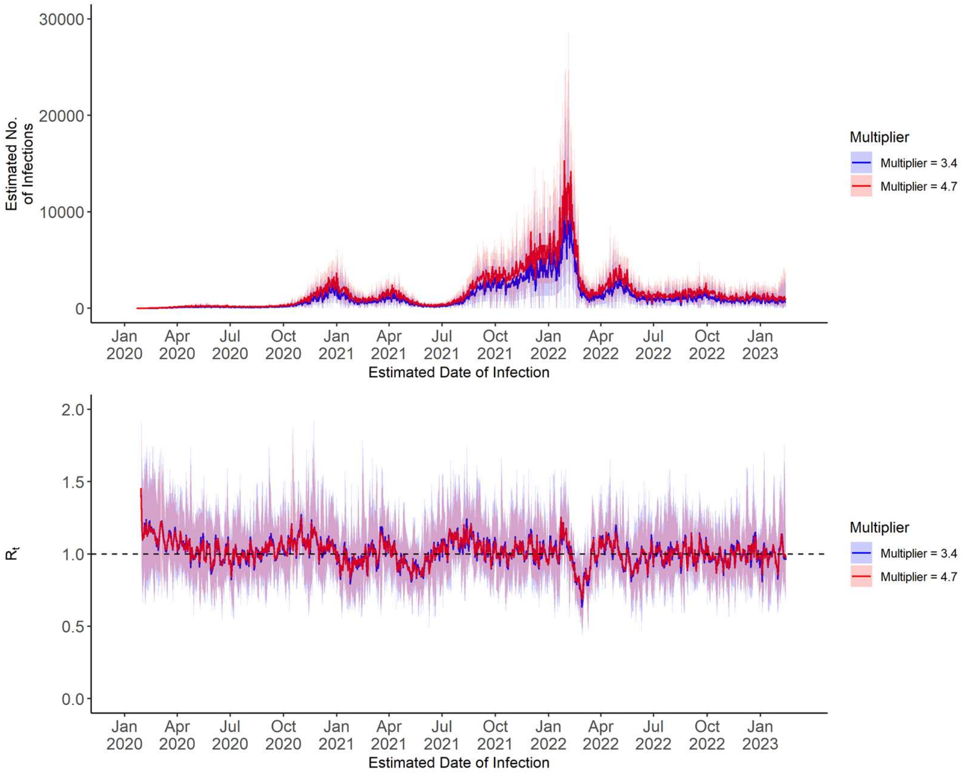

Figure S6. Changes in Rt following major policy interventions for Maine statewide and its 16 counties in 2020. Labels: A – Stay Healthy at Home Directive (April 2, 2020); B – Re-opening (Stage 1) (May 1, 2020); C – Gradual Easing of Restrictions (May 31, 2020); D – Face Covering Mandate (Certain Businesses) (July 8, 2020); E – School Re-opening (September 8, 2020); F – Re-opening (Stage 4) (October 13, 2020); G – Face Covering Mandate (All Public Settings) (November 4, 2020).

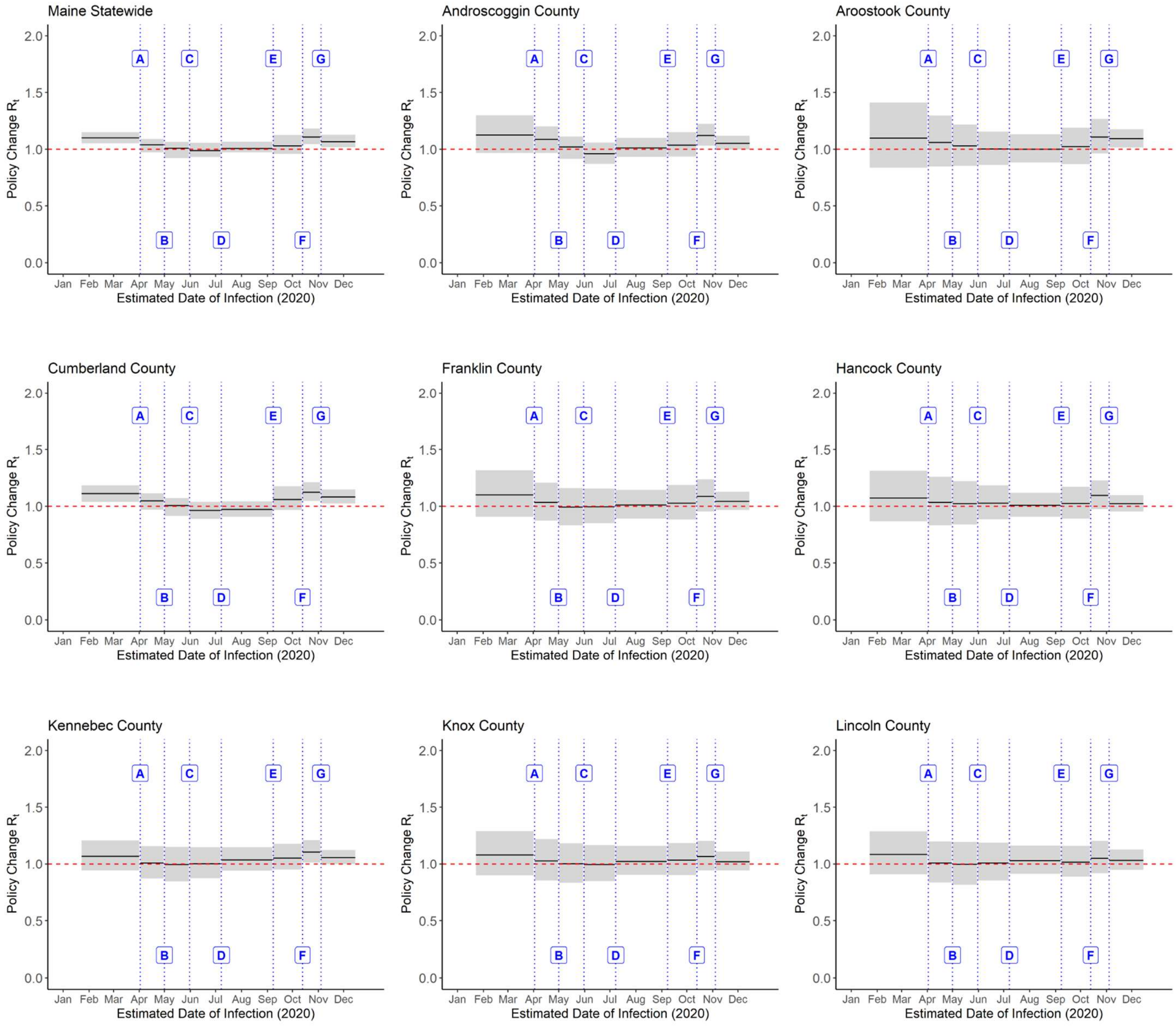

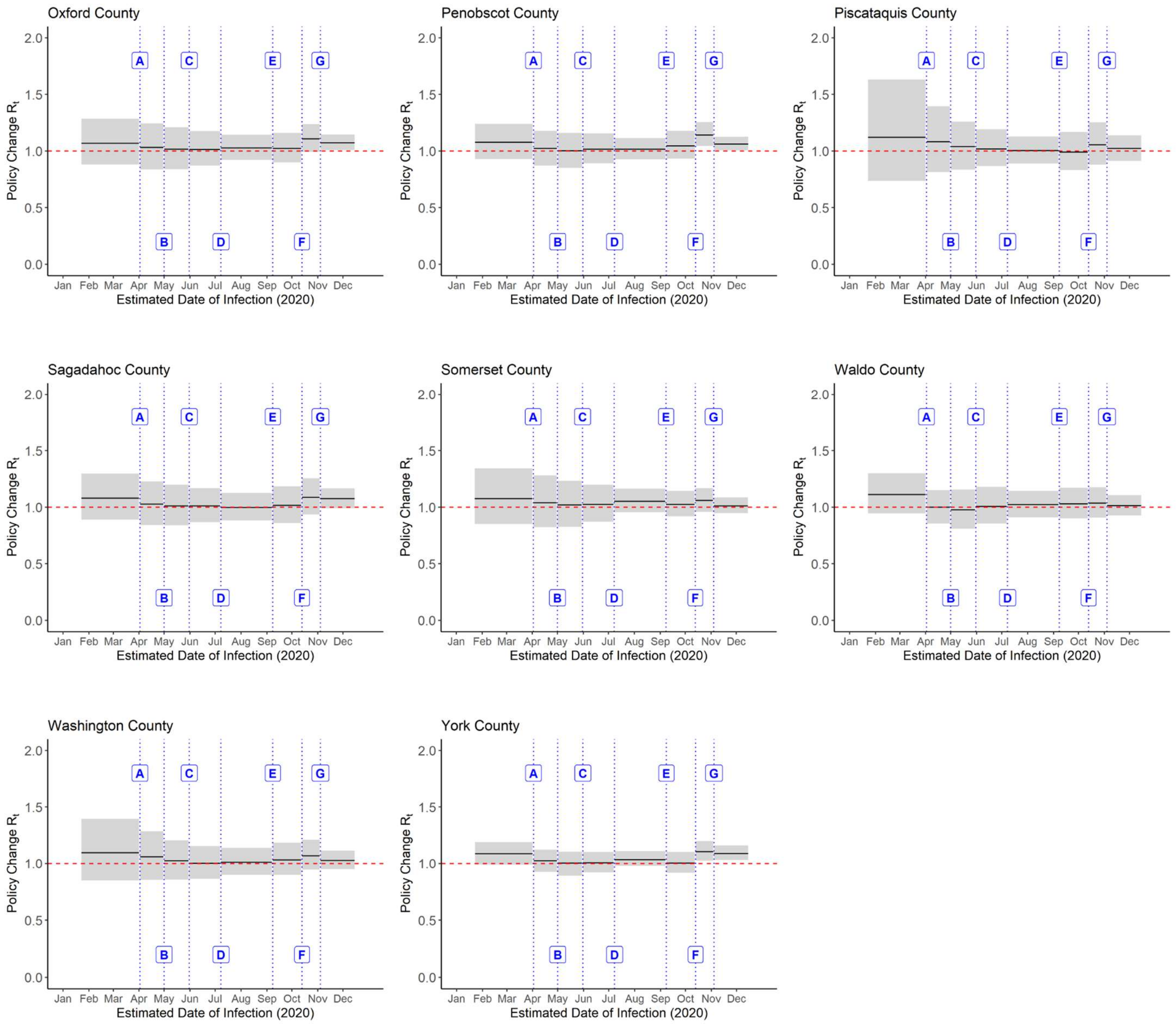
Oxford County
Penobscot County
Piscataquis County
Sagadahoc County
Somerset County
Waldo County
Washington County
York County
Policy Change R_t
2.0
1.5
1.0
0.5
0.0
A
B
C
D
E
F
G
Jan Feb Mar Apr May Jun Jul Aug Sep Oct Nov Dec
Estimated Date of Infection (2020)

Figure S7. Median percentage change and 95% CrI of Rt estimates associated with policy changes, for Maine statewide and its 16 counties. The x-axis represents the percentage change, with increases in Rt (positive values) shown to the right of the vertical dotted line at zero and decreases in Rt (negative values) shown to the left.

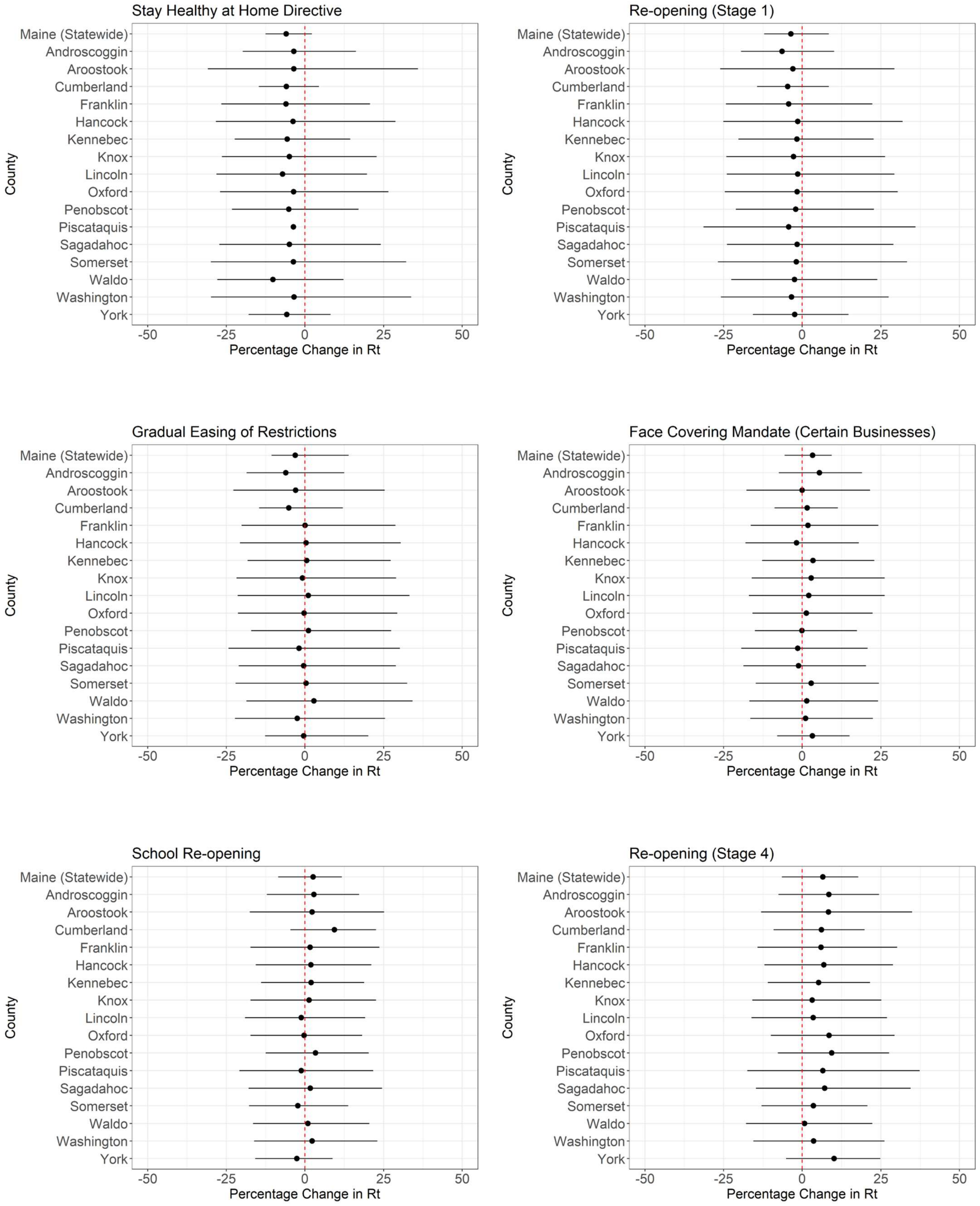

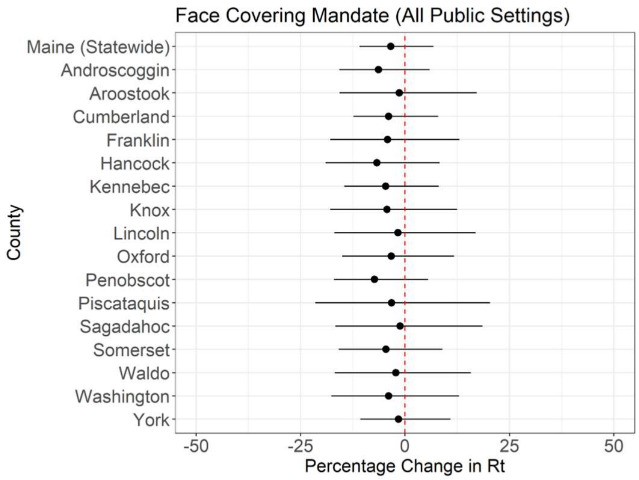
Face Covering Mandate (All Public Settings)
County
Maine (Statewide)
Androscoggin
Aroostook
Cumberland
Franklin
Hancock
Kennebec
Knox
Lincoln
Oxford
Penobscot
Piscataquis
Sagadahoc
Somerset
Waldo
Washington
York
-50
-25
0
25
50
Percentage Change in Rt

References

1. State of Maine Office of Governor Janet T. Mills. Governor Mills convenes coronavirus response team. Available online: https://www.maine.gov/governor/mills/news/governor-mills-convenes-coronavirus-response-team-2020-03-02 (accessed on December 15, 2024).
2. State of Maine Office of Governor Janet T. Mills. Governor Mills, Maine CDC announce first presumptive positive case and additional steps to respond to COVID-19. Available online: https://www.maine.gov/governor/mills/news/governor-mills-maine-cdc-announce-first-presumptive-positive-case-and-additional-steps-respond (accessed on December 15, 2024).
3. State of Maine Office of Governor Janet T. Mills. Governor Mills takes step to protect Maine workers and small businesses impacted by coronavirus. Available online: https://www.maine.gov/governor/mills/news/governor-mills-takes-step-protect-maine-workers-small-businesses-impacted-coronavirus-2020-03 (accessed on December 15, 2024).
4. State of Maine Office of Governor Janet T. Mills. Governor announces significant recommendations and signs civil emergency proclamation to respond to COVID-19 in Maine. Available online: https://www.maine.gov/governor/mills/news/governor-announces-significant-recommendations-signs-civil-emergency-proclamation-respond (accessed on December 15, 2024).
5. State of Maine Office of Governor Janet T. Mills. Governor Mills urges cancellation of Saint Patrick's Day events statewide. Available online: https://www.maine.gov/governor/mills/news/governor-mills-urges-cancellation-saint-patricks-day-events-statewide-2020-03-16 (accessed on December 15, 2024).
6. State of Maine Office of Governor Janet T. Mills. Governor Mills, legislative leaders reach bipartisan agreement on revised supplemental budget proposal. Available online: https://www.maine.gov/governor/mills/news/governor-mills-legislative-leaders-reach-bipartisan-agreement-revised-supplemental-budget (accessed on December 15, 2024).
7. State of Maine Office of Governor Janet T. Mills. Governor Mills issues declaration to protect Maine people against price-gouging. Available online: https://www.maine.gov/governor/mills/news/governor-mills-issues-declaration-protect-maine-people-against-price-gouging-2020-03-17 (accessed on December 15, 2024).
8. State of Maine Office of Governor Janet T. Mills. Governor Mills, legislature spearhead emergency legislative package to respond to COVID-19. Available online: https://www.maine.gov/governor/mills/news/governor-mills-legislature-spearhead-emergency-legislative-package-respond-covid-19-2020-03-17 (accessed on December 15, 2024).
9. State of Maine Office of Governor Janet T. Mills. Governor Mills takes further steps to respond to COVID-19, protect health and safety of Maine people. Available online: https://www.maine.gov/governor/mills/news/governor-mills-takes-further-steps-respond-covid-19-protect-health-and-safety-maine-people (accessed on December 15, 2024).
10. State of Maine Office of Governor Janet T. Mills. Governor Mills presses federal government to provide Maine with more personal protective equipment and testing supplies. Available online: https://www.maine.gov/governor/mills/news/governor-mills-presses-federal-government-provide-maine-more-personal-protective-equipment-and (accessed on December 15, 2024).
11. State of Maine Office of Governor Janet T. Mills. Governor Mills, Maine congressional delegation convene call on COVID-19. Available online: https://www.maine.gov/governor/mills/news/governor-mills-maine-congressional-delegation-convene-call-covid-19-2020-03-19 (accessed on December 15, 2024).
12. State of Maine Office of Governor Janet T. Mills. An order regarding school readiness. Available online: https://www.maine.gov/governor/mills/sites/maine.gov.governor.mills/files/inline-files/EO%2015%20An%20Order%20Regarding%20School%20Readiness.pdf (accessed on December 15, 2024).
13. State of Maine Office of Governor Janet T. Mills. Governor Mills, Commissioner Camuso suspend inland waters fishing license requirement, open waters to inland fishing. Available online: https://www.maine.gov/governor/mills/news/governor-mills-commissioner-camuso-suspend-inland-waters-fishing-license-requirement-open (accessed on December 15, 2024).

14. State of Maine Office of Governor Janet T. Mills. An order suspending provisions of certain health care professional licensing statutes and rules in order to facilitate the treatment and containment of COVID-19. Available online: https://www.maine.gov/governor/mills/sites/maine.gov.governor.mills/files/inline-files/EO%2016%20An%20Order%20Suspending%20Provisions%20of%20Certain%20HC%20Professional%20Licensing.pdf (accessed on December 15, 2024).
15. State of Maine Office of Governor Janet T. Mills. An order extending compliance dates under certain motor vehicle laws. Available online: https://www.maine.gov/governor/mills/sites/maine.gov.governor.mills/files/inline-files/EO%2018%20An%20Order%20Extending%20Compliance%20Dates%20Under%20Certain%20Motor%20Vehicle%20Laws.pdf (accessed on December 15, 2024).
16. State of Maine Office of Governor Janet T. Mills. Governor Mills orders further steps to protect public health. Available online: https://www.maine.gov/governor/mills/news/governor-mills-orders-further-steps-protect-public-health-2020-03-24 (accessed on December 15, 2024).
17. State of Maine Office of Governor Janet T. Mills. Mills administration, public higher education systems coordinating response to COVID-19. Available online: https://www.maine.gov/governor/mills/news/mills-administration-public-higher-education-systems-coordinating-response-covid-19-2020-03-25 (accessed on December 15, 2024).
18. State of Maine Office of Governor Janet T. Mills. Governor Mills acts to promote access to health care during COVID-19. Available online: https://www.maine.gov/governor/mills/news/governor-mills-acts-promote-access-health-care-during-covid-19-2020-03-25 (accessed on December 15, 2024).
19. State of Maine Office of Governor Janet T. Mills. Mills administration takes steps to support nursing homes in response to COVID-19. Available online: https://www.maine.gov/governor/mills/news/mills-administration-takes-steps-support-nursing-homes-response-covid-19-2020-03-26 (accessed on December 15, 2024).
20. State of Maine Office of Governor Janet T. Mills. Governor Mills extends state income tax payment deadline to July 15, 2020. Available online: https://www.maine.gov/governor/mills/news/governor-mills-extends-state-income-tax-payment-deadline-july-15-2020-2020-03-26 (accessed on December 15, 2024).
21. State of Maine Office of Governor Janet T. Mills. Select coastal state parks closed due to overcrowding. Available online: https://www.maine.gov/governor/mills/news/select-coastal-state-parks-closed-due-overcrowding-2020-03-26 (accessed on December 15, 2024).
22. State of Maine Office of Governor Janet T. Mills. Governor Mills launches new online resource outlining ways Mainers can help Mainers during COVID-19 pandemic. Available online: https://www.maine.gov/governor/mills/news/governor-mills-launches-new-online-resource-outlining-ways-mainers-can-help-mainers-during (accessed on December 15, 2024).
23. State of Maine Office of Governor Janet T. Mills. Governor Mills, Commissioner Camuso extend boating registration renewal period. Available online: https://www.maine.gov/governor/mills/news/governor-mills-commissioner-camuso-extend-boating-registration-renewal-period-2020-03-27 (accessed on December 15, 2024).
24. State of Maine Office of Governor Janet T. Mills. Governor Mills signs executive order to fast track free online job training at Maine's community colleges. Available online: https://www.maine.gov/governor/mills/news/governor-mills-signs-executive-order-fast-track-free-online-job-training-maines-community (accessed on December 15, 2024).
25. State of Maine Office of Governor Janet T. Mills. Mills administration takes steps to support personal care workers, Maine seniors in response to COVID-19. Available online: https://www.maine.gov/governor/mills/news/mills-administration-takes-steps-support-personal-care-workers-maine-seniors-response-covid-19 (accessed on December 15, 2024).
26. State of Maine Office of Governor Janet T. Mills. Governor Mills requests major disaster declaration from the federal government. Available online: https://www.maine.gov/governor/mills/news/governor-mills-requests-major-disaster-declaration-federal-government-2020-04-01 (accessed on December 15, 2024).

27. State of Maine Office of Governor Janet T. Mills. Governor Mills extends state's property tax exemption deadline. Available online: https://www.maine.gov/governor/mills/news/governor-mills-extends-states-property-tax-exemption-deadline-2020-04-01 (accessed on December 15, 2024).
28. State of Maine Office of Governor Janet T. Mills. Governor Mills issues stay healthy at home mandate. Available online: https://www.maine.gov/governor/mills/news/governor-mills-issues-stay-healthy-home-mandate-2020-03-31 (accessed on December 15, 2024).
29. State of Maine Office of Governor Janet T. Mills. Governor Mills issues executive order mandating quarantine restrictions on travelers arriving in Maine to protect public health and safety. Available online: https://www.maine.gov/governor/mills/news/governor-mills-issues-executive-order-mandating-quarantine-restrictions-travelers-arriving (accessed on December 15, 2024).
30. State of Maine Office of Governor Janet T. Mills. Governor Mills announces approval of federal disaster declaration for COVID-19 response in Maine. Available online: https://www.maine.gov/governor/mills/news/governor-mills-announces-approval-federal-disaster-declaration-covid-19-response-maine-2020-04 (accessed on December 15, 2024).
31. State of Maine Office of Governor Janet T. Mills. Governor Mills issues executive order to expand access to health care during COVID-19. Available online: https://www.maine.gov/governor/mills/news/governor-mills-issues-executive-order-expand-access-health-care-during-covid-19-2020-04-07 (accessed on December 15, 2024).
32. State of Maine Office of Governor Janet T. Mills. Governor Mills announces plan to open alternative care sites in coordination with Maine hospitals and cities of Portland and Bangor. Available online: https://www.maine.gov/governor/mills/news/governor-mills-announces-plan-open-alternative-care-sites-coordination-maine-hospitals-cities (accessed on December 15, 2024).
33. Maine Department of Education. Maine DOE delivers donated devices to Piscataquis County schools. Available online: https://mainedoenews.net/2020/04/09/media-release-maine-doe-delivers-donated-devices-to-piscataquis-county-schools/ (accessed on December 15, 2024).
34. State of Maine Office of Governor Janet T. Mills. Governor Mills signs executive order allowing remote notarizations. Available online: https://www.maine.gov/governor/mills/news/governor-mills-signs-executive-order-allowing-remote-notarizations-2020-04-08 (accessed on December 15, 2024).
35. State of Maine Office of Governor Janet T. Mills. Governor Mills issues executive order moving primary election to July 14th. Available online: https://www.maine.gov/governor/mills/news/governor-mills-issues-executive-order-moving-primary-election-july-14th-2020-04-10 (accessed on December 15, 2024).
36. State of Maine Office of Governor Janet T. Mills. Mills administration steps up support for Maine people with substance use disorder in response to COVID-19. Available online: https://www.maine.gov/tools/whatsnew/index.php?topic=DHS+Press+Releases&id=2363628&v=article (accessed on December 15, 2024).
37. State of Maine Office of Governor Janet T. Mills. Startups step up. Available online: https://www.maine.gov/decd/sites/maine.gov.decd/files/inline-files/Startup%20Step%20up%20Outreach%204.13.2020%20%28002%29.pdf (accessed on December 15, 2024).
38. State of Maine Office of Governor Janet T. Mills. Governor Mills extends state of civil emergency as Maine continues to combat COVID-19 (April 14, 2020). Available online: https://www.maine.gov/governor/mills/news/governor-mills-extends-state-civil-emergency-maine-continues-combat-covid-19-2020-04-14 (accessed on December 15, 2024).
39. Maine Department of Labor. Claimants will see additional $600 early next week, call-in times extended. Available online: https://www.maine.gov/labor/news_events/article.shtml?id=2381967 (accessed on December 15, 2024).
40. State of Maine Office of Governor Janet T. Mills. Proclamation to renew the state of civil emergency. Available online: https://www.maine.gov/governor/mills/sites/maine.gov.governor.mills/files/inline-files/Proclamation%20to%20Renew%20the%20State%20of%20Civil%20Emergency.pdf (accessed on December 15, 2024).

41. State of Maine Office of Governor Janet T. Mills. An order regarding unlawful evictions, writs of possession, and initiation of eviction proceedings. Available online: https://www.maine.gov/governor/mills/sites/maine.gov.governor.mills/files/inline-files/An%20Order%20Regarding%20Unlawful%20Evictions%2C%20Writs%20of%20Possession%2C%20and%20Initiation%20of%20Eviction%20Proceedings_0.pdf (accessed on December 15, 2024).
42. Maine State Housing Authority. Emergency Rental Assistance Program. Available online: https://mainehousing.org/programs-services/rental/rentaldetail/covid-19-rental-relief-program (accessed on December 15, 2024).
43. State of Maine Office of Governor Janet T. Mills. "FrontLine WarmLine" launches to help Maine's health care workers and first responders manage emotional toll of COVID-19. Available online: https://www.maine.gov/tools/whatsnew/index.php?topic=DHS+Press+Releases&id=2417012&v=article (accessed on December 15, 2024).
44. Maine Department of Inland Fisheries and Wildlife. Governor, commissioner open turkey season early, temporarily suspend registration requirement for spring season only. Available online: https://www.maine.gov/ifw/news-events/single-release.html?id=2417018 (accessed on December 15, 2024).
45. State of Maine Office of Governor Janet T. Mills. Nearly $11 million in federal funds to support essential workers and child care providers in response to COVID-19. Available online: https://www.maine.gov/governor/mills/news/nearly-11-million-federal-funds-support-essential-workers-and-child-care-providers-response (accessed on December 15, 2024).
46. State of Maine Office of Governor Janet T. Mills. Governor Mills outlines vision for a gradual, safe reopening of Maine's economy. Available online: https://www.maine.gov/governor/mills/news/governor-mills-outlines-vision-gradual-safe-reopening-maines-economy-2020-04-23 (accessed on December 15, 2024).
47. State of Maine Office of Governor Janet T. Mills. Governor Mills announces Consensus Economic Forecasting Commission, Revenue Forecasting Committee to convene early to assess economic ramifications of COVID-19 on state revenues. Available online: https://www.maine.gov/governor/mills/news/governor-mills-announces-consensus-economic-forecasting-commission-revenue-forecasting (accessed on December 15, 2024).
48. State of Maine Office of Governor Janet T. Mills. Governor Mills presents safe, gradual plan to restart Maine's economy. Available online: https://www.maine.gov/governor/mills/news/governor-mills-presents-safe-gradual-plan-restart-maines-economy-2020-04-28 (accessed on December 15, 2024).
49. State of Maine Office of Governor Janet T. Mills. Governor Mills extends Maine's stay at home order. Available online: https://www.maine.gov/governor/mills/news/governor-mills-extends-maines-stay-home-order-2020-04-30 (accessed on December 15, 2024).
50. State of Maine Office of Governor Janet T. Mills. Governor Mills convenes expert committee to advise on state's economic recovery. Available online: https://www.maine.gov/governor/mills/news/governor-mills-convenes-expert-committee-advise-states-economic-recovery-2020-05-06-0 (accessed on December 15, 2024).
51. State of Maine Office of Governor Janet T. Mills. Mills administration secures major COVID-19 testing expansion for Maine. Available online: https://www.maine.gov/governor/mills/news/mills-administration-secures-major-covid-19-testing-expansion-maine-2020-05-07 (accessed on December 15, 2024).
52. State of Maine Office of Governor Janet T. Mills. Governor Mills introduces rural reopening plan. Available online: https://www.maine.gov/governor/mills/news/governor-mills-introduces-rural-reopening-plan-2020-05-08 (accessed on December 15, 2024).
53. State of Maine Office of Governor Janet T. Mills. Launch of a statewide campaign to promote awareness of affordable health insurance options. Available online: https://www.maine.gov/tools/whatsnew/index.php%3Ftopic%3DDHS%2BPress%2BReleases%26id%3D2529309%26v%3Darticle (accessed on December 15, 2024).

54. State of Maine Office of Governor Janet T. Mills. Governor Mills extends state of civil emergency as Maine continues to combat COVID-19 (May 13, 2020). Available online: https://www.maine.gov/governor/mills/news/governor-mills-extends-state-civil-emergency-maine-continues-combat-covid-19-2020-05-13 (accessed on December 15, 2024).
55. State of Maine Office of Governor Janet T. Mills. Mills administration allows lodging reservations for future stays. Available online: https://www.maine.gov/governor/mills/news/mills-administration-allows-lodging-reservations-future-stays-2020-05-14 (accessed on December 15, 2024).
56. State of Maine Office of Governor Janet T. Mills. Mills administration secures WiFi and learning devices for 100 percent of Maine students reporting a need in face of COVID-19's impacts on schools. Available online: https://www.maine.gov/governor/mills/news/mills-administration-secures-wifi-learning-devices-100-percent-maine-students-reporting-need (accessed on December 15, 2024).
57. State of Maine Office of Governor Janet T. Mills. Maine Emergency Management Agency (MEMA) delivers cloth face coverings to Maine Association of Broadcasters for distribution to essential media workers. Available online: https://www.maine.gov/governor/mills/news/maine-emergency-management-agency-mema-delivers-cloth-face-coverings-maine-association (accessed on December 15, 2024).
58. State of Maine Office of Governor Janet T. Mills. Mills administration updates plan to restart Maine's economy. Available online: https://www.maine.gov/governor/mills/news/mills-administration-updates-plan-restart-maines-economy-2020-05-19 (accessed on December 15, 2024).
59. State of Maine Office of Governor Janet T. Mills. Governor Mills announces new major federal funding will be used to expand lab and testing capacity across Maine. Available online: https://www.maine.gov/governor/mills/news/governor-mills-announces-new-major-federal-funding-will-be-used-expand-lab-and-testing (accessed on December 15, 2024).
60. State of Maine Office of Governor Janet T. Mills. Maine expands contact tracing to limit the spread of COVID-19. Available online: https://www.maine.gov/governor/mills/news/maine-expands-contact-tracing-limit-spread-covid-19-2020-05-26 (accessed on December 15, 2024).
61. State of Maine Office of Governor Janet T. Mills. Mills administration announces update to Restarting Maine's Economy plan. Available online: https://www.maine.gov/governor/mills/news/mills-administration-announces-update-restarting-maines-economy-plan-2020-05-27 (accessed on December 15, 2024).
62. State of Maine Office of Governor Janet T. Mills. Governor Mills issues executive order allowing for easing of restrictions as Restarting Maine plan progresses. Available online: https://www.maine.gov/governor/mills/news/governor-mills-issues-executive-order-allowing-easing-restrictions-restarting-maine-plan (accessed on December 15, 2024).
63. State of Maine Office of Governor Janet T. Mills. At direction of Governor Mills, Maine Department of Corrections to support restaurants impacted by dine-in postponement. Available online: https://www.maine.gov/governor/mills/news/direction-governor-mills-maine-department-corrections-support-restaurants-impacted-dine (accessed on December 15, 2024).
64. Maine State Housing Authority. Governor Mills and MaineHousing extend rent relief program. Available online: https://www.mainehousing.org/news/news-detail/2020/06/04/governor-mills-mainehousing-extend-rent-relief-program (accessed on December 15, 2024).
65. State of Maine Office of Governor Janet T. Mills. In light of data trends, Mills administration accelerates business openings in rural counties. Available online: https://www.maine.gov/governor/mills/news/light-data-trends-mills-administration-accelerates-business-openings-rural-counties-2020-06-04 (accessed on December 15, 2024).
66. State of Maine Office of Governor Janet T. Mills. Mills administration announces vast expansion of COVID-19 testing. Available online: https://www.maine.gov/governor/mills/news/mills-administration-announces-vast-expansion-covid-19-testing-2020-06-08 (accessed on December 15, 2024).
67. State of Maine Office of Governor Janet T. Mills. Mills administration unveils "Keep Maine Healthy" plan to protect Maine people, visitors and support small businesses during tourism season. Available online:

https://www.maine.gov/governor/mills/news/mills-administration-unveils-keep-maine-healthy-plan-protect-maine-people-visitors-support (accessed on December 15, 2024).
68. State of Maine Office of Governor Janet T. Mills. Governor Mills renews state of civil emergency for 30 days as Maine reopens, continues to combat COVID-19. Available online: https://www.maine.gov/governor/mills/news/governor-mills-renews-state-civil-emergency-30-days-maine-reopens-continues-combat-covid-19 (accessed on December 15, 2024).
69. State of Maine Office of Governor Janet T. Mills. Mills administration accelerates start date for lodging establishments to serve out-of-state visitors. Available online: https://www.maine.gov/governor/mills/news/mills-administration-accelerates-start-date-lodging-establishments-serve-out-state-visitors (accessed on December 15, 2024).
70. State of Maine Office of Governor Janet T. Mills. Maine DHHS awards CARES Act funding to support essential workers and child care providers in response to COVID-19. Available online: https://www.maine.gov/governor/mills/news/maine-dhhs-awards-cares-act-funding-support-essential-workers-and-child-care-providers (accessed on December 15, 2024).
71. State of Maine Office of Governor Janet T. Mills. Mills administration announces that indoor dining in Androscoggin, Cumberland and York counties can resume on June 17 with added health and safety precautions. Available online: https://www.maine.gov/governor/mills/news/mills-administration-announces-indoor-dining-androscoggin-cumberland-york-counties-can-resume (accessed on December 15, 2024).
72. State of Maine Office of Governor Janet T. Mills. Mills administration releases guidance for town meetings and elections. Available online: https://www.maine.gov/governor/mills/news/mills-administration-releases-guidance-town-meetings-and-elections-2020-06-15 (accessed on December 15, 2024).
73. State of Maine Office of Governor Janet T. Mills. Governor Mills urges USDA to add maple syrup to the Coronavirus Food Assistance Program. Available online: https://www.maine.gov/governor/mills/news/governor-mills-urges-usda-add-maple-syrup-coronavirus-food-assistance-program-2020-06-22 (accessed on December 15, 2024).
74. State of Maine Office of Governor Janet T. Mills. To protect public health, Mills administration postpones reopening of indoor bar service across Maine. Available online: https://www.maine.gov/governor/mills/news/protect-public-health-mills-administration-postpones-reopening-indoor-bar-service-across-maine (accessed on December 15, 2024).
75. State of Maine Office of Governor Janet T. Mills. Mills administration dedicates Coronavirus Relief Funding for state and local match on FEMA public assistance. Available online: https://www.maine.gov/governor/mills/news/mills-administration-dedicates-coronavirus-relief-funding-state-and-local-match-fema-public (accessed on December 15, 2024).
76. State of Maine Office of Governor Janet T. Mills. Mills administration approves COVID-19 prevention and protection grant awards to municipalities as part of Keep Maine Healthy plan. Available online: https://www.maine.gov/governor/mills/news/mills-administration-approves-covid-19-prevention-and-protection-grant-awards-municipalities (accessed on December 15, 2024).
77. State of Maine Office of Governor Janet T. Mills. With improving public health metrics, Mills administration exempts Connecticut, New York, and New Jersey from quarantine and testing requirement. Available online: https://www.maine.gov/governor/mills/news/improving-public-health-metrics-mills-administration-exempts-connecticut-new-york-and-new (accessed on December 15, 2024).
78. State of Maine Office of Governor Janet T. Mills. To fight COVID-19, Governor Mills strengthens enforcement of face covering requirement as Maine reopens. Available online: https://www.maine.gov/governor/mills/news/fight-covid-19-governor-mills-strengthens-enforcement-face-covering-requirement-maine-reopens (accessed on December 15, 2024).
79. State of Maine Office of Governor Janet T. Mills. Mills administration announces nearly 20 "Swab and Send" COVID-19 testing sites. Available online: https://www.maine.gov/governor/mills/news/mills-administration-announces-nearly-20-swab-and-send-covid-19-testing-sites-2020-07-14 (accessed on December 15, 2024).
80. State of Maine Office of Governor Janet T. Mills. Economic Recovery Committee delivers recommendations to Governor Mills to stabilize businesses and support Maine people amidst pandemic. Available online:

https://www.maine.gov/governor/mills/news/economic-recovery-committee-delivers-recommendations-governor-mills-stabilize-businesses-and (accessed on December 15, 2024).
81. State of Maine Office of Governor Janet T. Mills. Mills administration supports child care for working families with $8 million investment from Coronavirus Relief Fund. Available online: https://www.maine.gov/governor/mills/news/mills-administration-supports-child-care-working-families-8-million-investment-coronavirus (accessed on December 15, 2024).
82. State of Maine Office of Governor Janet T. Mills. To protect health of students and staff, Mills administration provides public health guidance and financial support to school systems across Maine. Available online: https://www.maine.gov/governor/mills/news/protect-health-students-staff-mills-administration-provides-public-health-guidance-financial (accessed on December 15, 2024).
83. State of Maine Office of Governor Janet T. Mills. Maine DHHS announces four additional "Swab and Send" COVID-19 testing sites. Available online: https://www.maine.gov/governor/mills/news/maine-dhhs-announces-four-additional-swab-and-send-covid-19-testing-sites-2020-07-21 (accessed on December 15, 2024).
84. State of Maine Office of Governor Janet T. Mills. Investment of $1 million from the Coronavirus Relief Fund to expand services to help reduce racial and ethnic disparities. Available online: https://www.maine.gov/tools/whatsnew/index.php%3Ftopic%3DDHS%2BPress%2BReleases%26id%3D3005753%26v%3Ddhhs_article_2020 (accessed on December 15, 2024).
85. State of Maine Office of Governor Janet T. Mills. Governor Mills and MaineHousing expand rental relief program to help prevent evictions, counter reductions in federal unemployment benefits. Available online: https://www.maine.gov/governor/mills/news/governor-mills-mainehousing-expand-rental-relief-program-help-prevent-evictions-counter (accessed on December 15, 2024).
86. State of Maine Office of Governor Janet T. Mills. As Maine seeks to maintain relatively low virus spread, Governor Mills renews state of civil emergency. Available online: https://www.maine.gov/governor/mills/news/maine-seeks-maintain-relatively-low-virus-spread-governor-mills-renews-state-civil-emergency (accessed on December 15, 2024).
87. State of Maine Office of Governor Janet T. Mills. Mills administration approves second round of COVID-19 prevention and protection awards under Keep Maine Healthy plan. Available online: https://www.maine.gov/governor/mills/news/mills-administration-approves-second-round-covid-19-prevention-and-protection-awards-under (accessed on December 15, 2024).
88. State of Maine Office of Governor Janet T. Mills. Maine DHHS and MaineHealth announce five new "Swab and Send" COVID-19 testing sites opening next week. Available online: https://www.maine.gov/governor/mills/news/maine-dhhs-and-mainehealth-announce-five-new-swab-and-send-covid-19-testing-sites-opening-next (accessed on December 15, 2024).
89. State of Maine Office of Governor Janet T. Mills. Governor Mills launches $200 million economic recovery grant program to support Maine small businesses. Available online: https://www.maine.gov/governor/mills/news/governor-mills-launches-200-million-economic-recovery-grant-program-support-maine-small (accessed on December 15, 2024).
90. State of Maine Office of Governor Janet T. Mills. Governor Mills signs executive order to facilitate voting, protect health of voters and election officials amid COVID-19 pandemic. Available online: https://www.maine.gov/governor/mills/news/governor-mills-signs-executive-order-facilitate-voting-protect-health-voters-election (accessed on December 15, 2024).
91. State of Maine Office of Governor Janet T. Mills. As state monitors outbreaks and schools resume for fall, Governor Mills extends state of civil emergency. Available online: https://www.maine.gov/governor/mills/news/state-monitors-outbreaks-and-schools-resume-fall-governor-mills-extends-state-civil-emergency (accessed on December 15, 2024).
92. State of Maine Office of Governor Janet T. Mills. Governor Mills signs curtailment order to maintain budget stability amid COVID-19 pandemic. Available online: https://www.maine.gov/governor/mills/news/governor-mills-signs-curtailment-order-maintain-budget-stability-amid-covid-19-pandemic-2020 (accessed on December 15, 2024).

93. State of Maine Office of Governor Janet T. Mills. Governor Mills announces Phase 2 of Maine Economic Recovery Grant Program funding for expanded pool of Maine businesses and non-profits. Available online: https://www.maine.gov/governor/mills/news/governor-mills-announces-phase-2-maine-economic-recovery-grant-program-funding-expanded-pool (accessed on December 15, 2024).
94. State of Maine Office of Governor Janet T. Mills. Mills administration broadens standing order to allow anyone in Maine to get tested for COVID-19. Available online: https://www.maine.gov/governor/mills/news/mills-administration-broadens-standing-order-allow-anyone-maine-get-tested-covid-19-2020-09-22 (accessed on December 15, 2024).
95. State of Maine Office of Governor Janet T. Mills. To protect the health of students and staff, Governor Mills announces additional financial support for school systems across Maine. Available online: https://www.maine.gov/governor/mills/news/protect-health-students-and-staff-governor-mills-announces-additional-financial-support-school (accessed on December 15, 2024).
96. State of Maine Office of Governor Janet T. Mills. Travelers from Massachusetts are exempt from the requirement to get a negative COVID-19 test or quarantine for 14 days. Available online: https://www.maine.gov/tools/whatsnew/index.php%3Ftopic%3DDHS%2BPress%2BReleases%26id%3D3337525%26v%3Ddhhs_article_2020 (accessed on December 15, 2024).
97. State of Maine Office of Governor Janet T. Mills. Governor Mills continues state of civil emergency as Maine fights COVID-19 (September 30, 2020). Available online: https://www.maine.gov/governor/mills/news/governor-mills-continues-state-civil-emergency-maine-fights-covid-19-2020-09-30 (accessed on December 15, 2024).
98. State of Maine Office of Governor Janet T. Mills. Mills administration announces Maine to enter Stage 4 of reopening. Available online: https://www.maine.gov/governor/mills/news/mills-administration-announces-maine-enter-stage-4-reopening-2020-10-06 (accessed on December 15, 2024).
99. State of Maine Office of Governor Janet T. Mills. Governor Mills announces first round of economic recovery grants to Maine small businesses and non-profits. Available online: https://www.maine.gov/governor/mills/news/governor-mills-announces-first-round-economic-recovery-grants-maine-small-businesses-non (accessed on December 15, 2024).
100. State of Maine Office of Governor Janet T. Mills. Mills administration announces "OPTIONS" initiative to support Maine people with substance use disorder. Available online: https://www.maine.gov/governor/mills/news/mills-administration-announces-options-initiative-support-maine-people-substance-use-disorder (accessed on December 15, 2024).
101. State of Maine Office of Governor Janet T. Mills. Governor Mills continues state of civil emergency as Maine fights COVID-19 (October 29, 2020). Available online: https://www.maine.gov/governor/mills/news/governor-mills-continues-state-civil-emergency-maine-fights-covid-19-2020-10-29 (accessed on December 15, 2024).
102. State of Maine Office of Governor Janet T. Mills. Distribution of rapid COVID-19 antigen tests. Available online: https://www.maine.gov/tools/whatsnew/index.php%3Ftopic%3DDHS%2BPress%2BReleases%26id%3D3536670%26v%3Ddhhs_article_2020 (accessed on December 15, 2024).
103. State of Maine Office of Governor Janet T. Mills. With cases rising in Maine and nationwide, Mills administration announces measures to prevent spread of the deadly COVID-19 virus. Available online: https://www.maine.gov/governor/mills/news/cases-rising-maine-nationwide-mills-administration-announces-measures-prevent-spread-deadly (accessed on December 15, 2024).
104. State of Maine Office of Governor Janet T. Mills. Following record COVID-19 cases, Governor Mills announces new face covering executive order. Available online: https://www.maine.gov/governor/mills/news/following-record-covid-19-cases-governor-mills-announces-new-face-covering-executive-order (accessed on December 15, 2024).
105. State of Maine Office of Governor Janet T. Mills. Joint statement from the offices of governors Murphy, Mills, Raimondo, Sununu, Scott, Lamont, and Baker on the suspension of interstate youth hockey. Available online: https://www.maine.gov/governor/mills/news/joint-statement-offices-governors-murphy-mills-raimondo-sununu-scott-lamont-and-baker (accessed on December 15, 2024).

106. State of Maine Office of Governor Janet T. Mills. Ahead of holiday season, Mills administration announces Massachusetts no longer exempt from quarantine or negative test requirement. Available online: https://www.maine.gov/governor/mills/news/ahead-holiday-season-mills-administration-announces-massachusetts-no-longer-exempt-quarantine (accessed on December 15, 2024).
107. State of Maine Office of Governor Janet T. Mills. With COVID-19 cases rising, Mills administration announces more public health and safety measures. Available online: https://www.maine.gov/governor/mills/news/covid-19-cases-rising-mills-administration-announces-more-public-health-and-safety-measures (accessed on December 15, 2024).
108. State of Maine Office of Governor Janet T. Mills. Mills administration awards $5.6 million to build high-speed internet infrastructure for students in underserved Maine communities. Available online: https://www.maine.gov/governor/mills/news/mills-administration-awards-56-million-build-high-speed-internet-infrastructure-students (accessed on December 15, 2024).
109. State of Maine Office of Governor Janet T. Mills. Governor Mills announces second round of economic recovery grants to Maine small businesses and non-profit. Available online: https://www.maine.gov/governor/mills/news/governor-mills-announces-second-round-economic-recovery-grants-maine-small-businesses-non (accessed on December 15, 2024).
110. State of Maine Office of Governor Janet T. Mills. Governor Mills dedicates $6.2 million more to rent relief to support Maine families. Available online: https://www.maine.gov/governor/mills/news/governor-mills-dedicates-62-million-more-rent-relief-support-maine-families-2020-11-20 (accessed on December 15, 2024).
111. State of Maine Office of Governor Janet T. Mills. Governor Mills continues state of civil emergency as Maine fights COVID-19 (November 24, 2020). Available online: https://www.maine.gov/governor/mills/news/governor-mills-continues-state-civil-emergency-maine-fights-covid-19-2020-11-24 (accessed on December 15, 2024).
112. State of Maine Office of Governor Janet T. Mills. Mills administration invests $536,000 in Coronavirus Relief Funds for Meals on Wheels. Available online: https://www.maine.gov/governor/mills/news/mills-administration-invests-536000-coronavirus-relief-funds-meals-wheels-2020-11-25 (accessed on December 15, 2024).
113. State of Maine Office of Governor Janet T. Mills. Governor Mills launches $40 million economic recovery grant program to support Maine tourism, hospitality and retail small businesses. Available online: https://www.maine.gov/governor/mills/news/governor-mills-launches-40-million-economic-recovery-grant-program-support-maine-tourism (accessed on December 15, 2024).
114. State of Maine Office of Governor Janet T. Mills. Mills administration announces new grants for Maine health care organizations. Available online: https://www.maine.gov/governor/mills/news/mills-administration-announces-new-grants-maine-health-care-organizations-2020-11-30 (accessed on December 15, 2024).
115. State of Maine Office of Governor Janet T. Mills. Mills administration continues early business closing time amid rising cases, hospitalizations. Available online: https://www.maine.gov/governor/mills/news/mills-administration-continues-early-business-closing-time-amid-rising-cases-hospitalizations (accessed on December 15, 2024).
116. State of Maine Office of Governor Janet T. Mills. Mills administration dedicates $2 million to support Maine food banks and pantries. Available online: https://www.maine.gov/governor/mills/news/mills-administration-dedicates-2-million-support-maine-food-banks-and-pantries-2020-12-04 (accessed on December 15, 2024).
117. State of Maine Office of Governor Janet T. Mills. Mills administration dedicates $10 million to new program to assist Maine food and agriculture businesses with COVID-19 infrastructure expenses. Available online: https://www.maine.gov/governor/mills/news/mills-administration-dedicates-10-million-new-program-assist-maine-food-and-agriculture (accessed on December 15, 2024).
118. State of Maine Office of Governor Janet T. Mills. Maine Department of Marine Resources launches new Maine seafood brand initiative. Available online: https://www.maine.gov/governor/mills/news/maine-department-marine-resources-launches-new-maine-seafood-brand-initiative-2020-12-09 (accessed on December 15, 2024).

119. State of Maine Office of Governor Janet T. Mills. Mills administration announces one-time relief payment for Mainers unemployed due to COVID-19. Available online: https://www.maine.gov/governor/mills/news/mills-administration-announces-one-time-relief-payment-mainers-unemployed-due-covid-19-2020-12 (accessed on December 15, 2024).
120. State of Maine Office of Governor Janet T. Mills. Mills administration commits full $1.25 billion of federal Coronavirus Relief funding. Available online: https://www.maine.gov/governor/mills/news/mills-administration-commits-full-125-billion-federal-coronavirus-relief-funding-2020-12-11 (accessed on December 15, 2024).
121. State of Maine Office of Governor Janet T. Mills. Governor Mills issues executive order strengthening enforcement of face covering requirement in all indoor public spaces. Available online: https://www.maine.gov/governor/mills/news/governor-mills-issues-executive-order-strengthening-enforcement-face-covering-requirement-all (accessed on December 15, 2024).
122. State of Maine Office of Governor Janet T. Mills. Governor Mills continues state of civil emergency as Maine fights COVID-19 (December 22, 2020). Available online: https://www.maine.gov/governor/mills/news/governor-mills-continues-state-civil-emergency-maine-fights-covid-19-2020-12-22 (accessed on December 15, 2024).
123. State of Maine Office of Governor Janet T. Mills. Mills administration continues early business closing time amid increase in COVID-19 positivity rate and hospitalizations. Available online: https://www.maine.gov/governor/mills/news/mills-administration-continues-early-business-closing-time-amid-increase-covid-19-positivity (accessed on December 15, 2024).
124. State of Maine Office of Governor Janet T. Mills. Joint statement from the offices of governors Murphy, Mills, Raimondo, Sununu, Scott, Lamont, and Baker on extending the suspension of interstate youth hockey. Available online: https://www.maine.gov/governor/mills/news/joint-statement-offices-governors-murphy-mills-raimondo-sununu-scott-lamont-and-baker-0 (accessed on December 15, 2024).
